# Introducing CARESSER: A framework for in situ learning robot social assistance from expert knowledge and demonstrations

**DOI:** 10.1007/s11257-021-09316-5

**Published:** 2022-03-12

**Authors:** Antonio Andriella, Carme Torras, Carla Abdelnour, Guillem Alenyà

**Affiliations:** 1 CSIC-UPC, Institut de Robòtica i Informàtica Industrial, C/Llorens i Artigas 4-6, 08028 Barcelona, Spain; 2 Research Center and Memory Clinic, Fundació ACE, Institut Català de Neurociències Aplicades, Universitat Internacional de Catalunya, Barcelona, Spain

**Keywords:** Robot adaptivity, Robot personalisation, Human–robot interaction, Robot-assisted cognitive training, Socially assistive robotics, In situ learning

## Abstract

Socially assistive robots have the potential to augment and enhance therapist’s effectiveness in repetitive tasks such as cognitive therapies. However, their contribution has generally been limited as domain experts have not been fully involved in the entire pipeline of the design process as well as in the automatisation of the robots’ behaviour. In this article, we present aCtive leARning agEnt aSsiStive bEhaviouR (CARESSER), a novel framework that actively learns robotic assistive behaviour by leveraging the therapist’s expertise (knowledge-driven approach) and their demonstrations (data-driven approach). By exploiting that hybrid approach, the presented method enables in situ fast learning, in a fully autonomous fashion, of personalised patient-specific policies. With the purpose of evaluating our framework, we conducted two user studies in a daily care centre in which older adults affected by mild dementia and mild cognitive impairment (*N* = 22) were requested to solve cognitive exercises with the support of a therapist and later on of a robot endowed with CARESSER. Results showed that: (i) the robot managed to keep the patients’ performance stable during the sessions even more so than the therapist; (ii) the assistance offered by the robot during the sessions eventually matched the therapist’s preferences. We conclude that CARESSER, with its stakeholder-centric design, can pave the way to new AI approaches that learn by leveraging human–human interactions along with human expertise, which has the benefits of speeding up the learning process, eliminating the need for the design of complex reward functions, and finally avoiding undesired states.

## Introduction

The incidence of cognitive disability has soared in the last decade, and it is projected to rise even further in the next 20 years (World Health Organization 2017). Rehabilitation robots can be a very useful tool to augment the effectiveness of therapists and to reduce their workload (Matarić 2017). Additionally, they can help to bridge the gap between the demands of the healthcare system and the shortage of healthcare professionals (Riek 2017; Abdi et al. 2018). This particularly applies to repetitive tasks. Indeed, cognitive and physical therapies can be dealt with by social robots. Furthermore, robots can offer their services at any time, without showing any form of boredom or tiredness.

Yet, how to replicate therapists’ expertise in terms of social intelligence in a robot in a fully autonomous fashion is still an open research question (Winkle et al. 2020). In order to address it, a robot needs to meet the following prerequisites (Senft et al. 2019): (i) its behaviour must be reasonable and understandable and not harm or undermine the person with whom it is interacting; (ii) it must learn quickly, as the number of interactions is usually very limited; (iii) it must be easy to set up and not require any technical expertise. To these requirements, we add the next one: (iv) it needs to lessen therapists’ workload.

In previous studies, three main methodologies have been adopted to learn a behaviour for the robot: Reinforcement Learning (RL) (Park et al. 2019; Clabaugh et al. 2019), Interactive Reinforcement Learning (IntRL) (Cruz et al. 2016; Amershi et al. 2014; Thomaz et al. 2005), and Learning from Demonstrations (LfD) (Knox et al. 2016; Liu et al. 2016). However, none of these approaches complies with all the four prerequisites above. RL is the most commonly used approach in SAR for learning robots’ behaviour; however, learning might be slow and designing a unique reward function that fits every individual’s special needs can be challenging. IntRL seems to speed up the learning process giving control of the robot’s learning behaviour to an expert. However, it still puts a huge burden on the expert whose reward signals might become inconsistent over time as they adapt their strategy. Finally, LfD via inverse reinforcement learning (IRL) addresses the main issue of RL by not requiring the design of any reward function, as the main goal of LfD is to estimate it from demonstrations. Nonetheless, employing them in real-world settings might require continuous demonstrations from the expert in order to adapt the robot’s behaviour to the changes in the environment. A recent method introduced by Senft et al. (2017), called SPARC, addressed three of the four aforementioned requirements. The method based on IntRL demonstrated in a user study its capability for generating autonomous robot behaviour, albeit the authors did not find any significant reduction in the therapist’s burden. Therefore, they only partially satisfy the last requirement we introduced. Furthermore, their authors did not consider adding prior knowledge of the human the robot is going to interact with (Petric and Kovacic 2020). In assistive scenarios, the human expert is usually the therapist, who knows their patients very well. Therefore, initialising the robot’s behaviour using the expert’s knowledge of the patient’s cognitive abilities can help to speed up the learning process and offer therapists an easy way to tune the robot’s initial behaviour. This is especially true in situations in which the number of interactions is limited.

In this study, we propose an alternative approach that aims to address two main aspects: short-term adaptivity and personalisation of robot socially assistive behaviour. To do that, we introduce aCtive leARning agEnt aSsiStive bEhaviouR (CARESSER), an interactive framework that enables robots to actively learn from both patients and therapists. CARESSER overcomes the issue related to the lack of data, leveraging the therapist’s prior knowledge of patients’ cognitive skills along with data gathered during daily therapies. To achieve that, a patient-specific simulator, which models the patient and the robot at symbolic high-level, was employed to generate data. As a result, CARESSER is capable of providing, at a given state of a task, tailored assistance to a specific patient, combining multi-modal interactions based on their individual needs.

In order to evaluate the proposed framework, we adopt a user-centred design approach, in which stakeholders (therapists, psychologists, and neurologists) collaborate in the design of a set of cognitive exercises aimed at training patients’ cognitive abilities (end users), such as memory and attention, along with motor functions, such as grasping. Thus, as a further benefit, stakeholders remain involved in the process of defining the requirements for developing the robot as well as its evaluation.

**Fig. 1:**
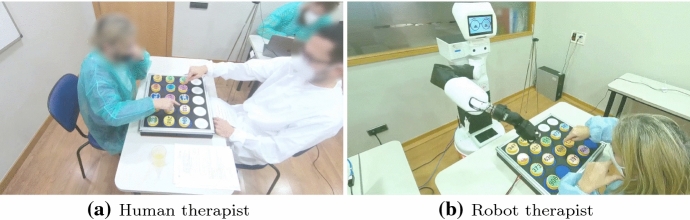
Example of a cognitive exercise session in which a therapist (either human or robot) assists a patient while playing a cognitive exercise

Firstly, we conduct an observational study in which a therapist is asked to provide clues and encouragement to a patient, in a multi-modal interaction fashion, during a cognitive exercise. In such a way, we inspect the therapist’s behaviour, namely the levels of assistance provided along with the patients’ performance with respect to several cognitive exercises. Secondly, we conduct a first user study (see Fig. 1a), in which a therapist is asked to interact with patients while they are playing a set of cognitive exercises, offering assistance according to the levels defined from the observational study. In such a manner, we gather data that would, later on, be used to initialise CARESSER. Thirdly, we carry out a second user study (see Fig. 1b) in which the therapist’s knowledge of the patients’ cognitive abilities, along with the gathered data from the previous study, is employed to produce a personalised patient-specific policy by leveraging CARESSER and evaluating it with a robot.

Ultimately, as to the best of our knowledge, this is the first study of a robot administering physical cognitive exercises in a fully autonomous manner to people affected by cognitive impairment, and we intend to shed some light from an AI perspective on the limitations of the approach and on what are the open challenges for roboticists that aim to deploy social robots for this type of population.

### Research questions

We consider an assistive scenario in which a robot is asked to learn how to provide, between several levels of assistance, those that best suit the individual’s specific needs on the basis of (i) the data collected from the interactions between the therapist and the patients on the same task; (ii) the a priori knowledge of the therapist about the patients’ cognitive capabilities; and (iii) the robot own interactions with the patients. Therefore, we raise the following question:

*RQ1*: Would the robot, endowed with CARESSER, be capable of providing adaptive and personalised assistance to a specific patient on the basis of their individual needs?

In order to properly address this research question, we reformulate it as follows:

*RQ1a*: Would the social assistance offered by the robot match the therapist’s preferences?
*RQ1b*: To what extent, if any, are the patients’ performances different when assisted by the robot therapist from when they are assisted by the human therapist or estimated by the patient-specific simulator?
*RQ1c*: Would the robot be able to keep the patient engaged to avoid both boredom and anxiety?

In RQ1a, we aim at evaluating whether the robot’s assistive behaviours are those expected by the human therapist. In RQ1b, we aim at assessing whether there is any difference between the patients’ performance when assisted by a human therapist, and when assisted by a robot. Additionally, whether the simulator, part of the CARESSER framework, is competent in modelling the patients’ cognitive abilities and generating data accordingly. Finally, in RQ1c, we aim at determining whether the robot therapist can keep the patients’ performances constant during the sessions. This last research question is based on the challenge point theory (Guadagnoli and Lee 2004) whereby optimal learning occurs when the task is neither too easy (boredom) nor too difficult (frustration).

### Rationale and contributions

We found there are at least two gaps in current research in: (i) designing and evaluating a socially assistive robot in a fully autonomous fashion for assisting patients with cognitive impairment and (ii) adapting the robot’s social behaviour to best suit the patient’s individual needs.

Regarding the design gap, some studies assumed what would be the functionalities of the robot, without considering stakeholders (including final users) and their specific needs (Hung et al. 2019). On the other hand, some studies focus on the patients, but the robotic platforms employed in the study lack significant capabilities to support the therapist and interact with the patients (Mancioppi et al. 2019). With respect to the level of autonomy of the robotic platform, the majority of the studies in real-world scenarios opted for a Wizard-of-Oz (WoZ) technique, in which robots are controlled by a human to make them appear as if they were autonomous in the “what, who, and when” offering assistance (Pino et al. 2020). This way, the robotic solution is far from being applicable. Finally, concerning the target population involved in the studies, very few of them were conducted with real patients; therefore, very little is known on how social robots work with vulnerable populations (Wang et al. 2017).

A second gap we identify is in the current methods for learning users’ preferences, which generally assume: (i) the access to a dataset or the possibility to easily gather data from interactions, (ii) the possibility to learn online, assuming a trial and error approach, (iii) the constant participation of a teacher or supervisor in the learning process, and (iv) the experimenter being able to design a reward function. In most real-world scenarios, like the one described here, these assumptions might fail.

In this study, we introduce CARESSER, with which we aim to address the issues mentioned above, with a special focus on the process of learning the different levels of assistance that best suit the patient’s individual needs. Specifically, the contributions of this work are the following:

- developing CARESSER, a framework that actively learns the robot’s socially assistive behaviours by leveraging therapist’s demonstrations and expertise,
- developing Generative mOdel Agent simuLation (GOAL), a patient-specific simulator which by means of generative Bayesian models of the patient and the robot keeps track of the patient’s cognitive abilities during the task and the robot’s assistive behaviour and generates interactions accordingly,
- designing effective robot’s socially assistive behaviours, which combine voice, gestures, and facial expressions, by involving stakeholders in the designing process,
- validating CARESSER in a fully autonomous robot with patients affected by mild cognitive impairment and mild dementia in a short-term in-situ cognitive training scenario.

### Challenges

In order to achieve the contributions above, we need to address several challenges:

- Modelling on a robot the therapist’s set of behaviours combining verbal and non-verbal social cues.
- Building a reliable cognitive model of the patient and the robot to simulate interactions and generate data.
- Learning a patient-specific policy from a limited number of interactions taking into account that the patient’s behaviour is extremely stochastic; thus, the system needs to keep track of it and adapt accordingly.
- Evaluating the fully autonomous robot in a real-world scenario with a vulnerable population.
- Using a physical board instead of a virtual board replicated on a device, which in turn implies no control over the users’ actions.

## Related work

Being able to provide tailored intervention has been demonstrated to be effective in the short- and long-term in situ assistive scenarios (Scassellati et al. 2018). Furthermore, it has been found that a suboptimal behaviour of the robot can affect the learning process (Kennedy et al. 2015). In order to achieve personalisation, several methodologies and approaches have been proposed. Here, we discuss the most relevant work divided by approach: Hidden Markov Model (HMM), Reinforcement Learning (RL), Interactive Reinforcement Learning (IntRL), Learning from Demonstration (LfD).

Bayesian Knowledge Tracing (BKT) and Bayesian Network (BN) are special types of HMM. Schodde et al. (2017) presented an approach based on BKT to personalising language tutoring in human–robot interaction. Results from a preliminary study indicated that participants learnt more successfully when interacting with a robot providing adaptive training compared to a robot providing random training. Leyzberg et al. (2014) designed a BN for skill assessment and evaluated whether and to what extent personalisation can affect students’ skills. Results from a long-term study revealed that first-grade students that received personalised assistance from Keapon outperformed students that received non-personalised lessons. A very inspirational work was done by Gordon and Breazeal (2015) who presented a social robot that, by employing a Bayesian active learning approach, allows the assessment of a child’s word-reading skills and adapts the interaction between robot and child to each child’s specific skill. Despite the short interaction, results showed the system could be personalised to different children’s ages and initial reading skills.

Compared to our work, these studies are mainly focused on teaching new skills to users, while we are more focused on training a specific cognitive ability. Similarly to our work, their approaches attempted to model the evolution of users’ skills (cognitive abilities) in an easy and understandable way. As Leyzberg et al. (2014), we employed a BN to model the patients’ cognitive abilities and the robot’s behaviour, and like Gordon and Breazeal (2015), our system actively updates its knowledge during the sessions. Differently, we adopt a hybrid approach to populate the BN combining real interactions with expert’s prior knowledge of the patients. Furthermore, we employed the Max Causal Entropy IRL algorithm (Ziebart 2010) for learning a reward function that encodes, in the form of features, information regarding the environment and the users and, thus, is capable of generating a more sophisticated robot’s policy.

A different approach, known as RL, is to leave the system to learn through experience by interacting with the environment and maximising its discounted expected future reward function. Chan and Nejat (2012) presented a robotic system called Brian 2.0 which was capable of engaging individuals in cognitively stimulating activities such as memory games. Another very interesting work was done by Moro et al. (2018). In their work, they combined LfD and RL to teach a robot socially assistive personalised behaviour. In order to validate their framework, the robot’s behaviours were taught by students, and the robot’s adaptivity was evaluated in simulation. Clabaugh et al. (2019) designed a socially assistive robot that could adapt and personalise its support to children with autism spectrum disorder during mathematical tasks. The results with 17 children showed that the robot achieved the objective of providing tailored assistance and, in addition, that it increased their engagement during the tasks. Similarly, Park et al. (2019) and Gordon et al. (2016) designed a SAR capable of tailoring its assistance to children learning literacy and a second language, respectively. Results showed that in the personalised condition, the participants could perform better in terms of learning, engagement, and word retaining.

Similar to our work,  Chan and Nejat (2012) and Moro et al. (2018) focus on creating a robot’s initial behaviour by modelling the users’ behaviour according to previously gathered data using a WoZ approach (Chan and Nejat 2012) or by assuming different users’ profiles (Moro et al. 2018). Differently, from Chan and Nejat (2012) and Moro et al. (2018), our simulator updates the user model after each session played by the robot with the patient in order to always have a reliable estimation of their capabilities. Furthermore, our user cognitive model is initialised with real data captured during those interactions and not just estimated. As Clabaugh et al. (2019), we evaluated our system in a real-world scenario with a vulnerable population, in our case, patients with cognitive impairment. In contrast to Clabaugh et al. (2019), Park et al. (2019) and Gordon et al. (2016), our robot starts interacting with the users given an initial behaviour defined by the gathered data from human–human interaction and the therapist’s expertise, potentially avoiding the initial exploration that might lead the robot into undesirable states.

With respect to our work, these studies showed how RL can be an effective approach for personalising human–robot interaction. However, RL requires the design of a reward function that is based on the assumption of “one size fits all”; that is, the same function should work for all the users. When the target population is people with special needs, such as older adults with cognitive impairment, it is very difficult to design a reward that works for all of them. Furthermore, its design can be very challenging when we try to integrate very heterogeneous information coming from multi-modal sensors (engagement level vs performance vs stress). Finally, RL requires a considerable amount of data to converge to an acceptable policy.

IntRL is based on the same fundamentals as RL, with the only exception that in this case, an expert, which can be either an agent or a human, can provide feedback or guidance and therefore reshape either the reward function, the action-value function, or the policy. Senft et al. (2019) presented a framework, called SPARC, that aimed to handover to an expert full control of the robot’s behaviour. Results from a user study with 75 participants revealed that SPARC was able to provide adaptive assistance and that it had an impact on the children in terms of learning gain. However, the decrease in the expert’s workload was not proved. Winkle et al. (2020) extended SPARC, providing it with the ability to personalise its behaviour to a specific individual. Preliminary results of a 9-week-long experiment highlighted that the robot could learn proper behaviour in a fully autonomous fashion, while it was not able to learn when offering assistance. Tsiakas et al. (2018) proposed an IntRL framework that was aimed at offering tailored assistance on the basis of individuals’ performance and their level of engagement during the task. The results provided evidence that when the feedback was employed, the task performance and the engagement of the simulated users increased.

From these studies, we see how IntRL seems a very promising approach for real-world scenarios as it reduces the number of interactions needed to learn reasonable assistive behaviour. Nonetheless, it requires the involvement of an expert for guiding the learning process during the whole duration of the task. This is the reason we decide to adopt a different approach in which the expert is only requested to initialise the system and then the robot will interact in a fully autonomous fashion.

LfD is one of the most efficient methods for transferring new skills to a machine by relying on demonstrations provided by a human. It is generally employed to learn low-level tasks, for instance, to learn a demonstrated motion trajectory. Very few works explored the opportunity of using it in high-level tasks, such as robot’s behaviour. Hussein et al. (2019) focused on learning the dynamics of interaction between a human and a robot. Results showed that the policies generated using the reward functions correctly mimic the demonstrator’s policies. Woodworth et al. (2018) presented a preference–inference formulation, in which a robot inferred a user’s preferences based only on observing the user’s behaviour in various tasks. Results suggested that the proposed algorithm, based on max-margin IRL, was capable of learning the user’s preferences during interactions with the robot. Sequeira et al. (2016) proposed a method for creating social interaction strategies for human–robot interaction based on WoZ studies. The final robot’s behaviours went through three design stages: data collection, in which the expert knowledge is gathered, strategy extraction, in which the robot’s strategy is learned from this data, and finally strategy refinement, in which the robot’s behaviour is interactively refined during the interactions. Similarly, Knox et al. (2014) presented a methodology to learn socially interactive behaviours using a WoZ paradigm. Louie and Nejat (2020) developed a system capable of learning new activities from non-expert teachers in order to autonomously facilitate therapeutic recreation interventions. A user study conducted in a residential care facility indicated that caregivers found the system easy to use and residents found the robot’s behaviour both pleasant and valuable.

In general, LfD either through human tele-operating a robot or performing the task themselves, has the main advantages of (i) avoiding inappropriate and low-quality behaviour that typically occurs in the early stages of an RL algorithm; (ii) being accessible to non-roboticist humans, as anyone can easily provide demonstrations to the robot. Furthermore, when the demonstrations are learnt by using IRL, there is no need to define any reward function as this is directly learnt from the demonstrations. This is very important to avoid a “one fits all approach”. These studies support our motivation for using LfD as a methodology to learn the therapist’s behaviour. Finally, all of these works provide solid foundations for understanding how adaptivity and personalisation can yield significant benefits in assistive human–robot interaction.

**Fig. 2:**
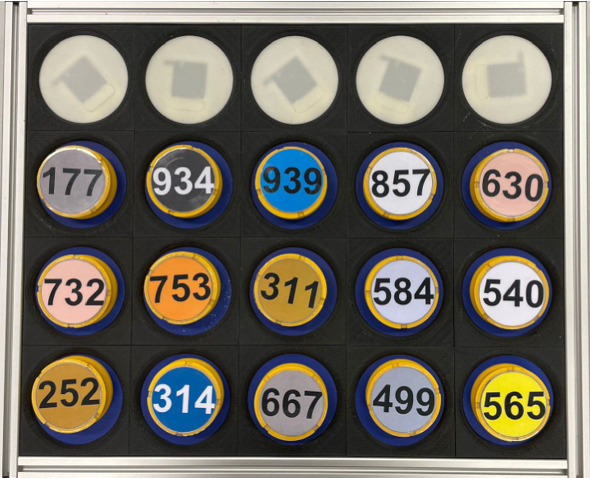
The electronic board employed during the experiment

## The cognitive training scenario

Cognitive stimulation, together with physical activity, is deemed to be among the most effective ways to reduce cognitive decline in later life. The vast majority of the work on social robots that assist individuals during cognitive tasks, e.g., cognitive exercises, employs electronic devices, in which the task is very often reduced to clicking on a screen. We argue that administering exercises via an electronic device deprives the interaction of one important component: the physical interaction with the objects in the space. Therefore, in collaboration with the healthcare professionals of the hospital, we proposed a set of cognitive exercises relying on the Syndrom-Kurztest (SKT) (Overall and Schaltenbrand 1992), which aimed not only to train memory and attention skills but also motor functions, such as grasping (De Boer et al. 2018). The SKT has been widely used to assess memory and attention deficits in individuals with cognitive impairment and dementia by means of a set of sub-tests. In some of these tests, patients are asked to sort tokens according to predefined criteria. Hence, we decided to include this type of exercise in our study similar to our previous works (Andriella et al. 2018, 2020b).

The criteria by which the tokens needed to be sorted were defined after several meetings with the stakeholders. Eight exercises were defined as follows:

- *sort_ascending*: sorting tokens in ascending order,
- *sort_descending*: sorting tokens in descending order,
- *sort_ascending_odd*: sorting odd tokens in ascending order,
- *sort_ascending_even*: sorting even tokens in ascending order,
- *sort_descending_odd*: sorting odd tokens in descending order,
- *sort_descending_even*: sorting even tokens in descending order,
- *sort_sum_ascending*: sorting tokens, based on the sum of their digits, in ascending order,
- *sort_sum_descending*: sorting tokens, based on the sum of their digits, in descending order.

Note that this was also the order of difficulty in which the exercises were classified. Note also that this setup allows other exercises, for example, using letters or pictures (Andriella et al. 2019c).

The board consists of twenty cells, five by four, and fifteen tokens randomly located in the second, third, and fourth rows, with plug and play numbers on the top (see Fig. 2). 180 numbers were available: 60 numbers between 1 and 99, 60 numbers between 100 and 999, and 60 numbers between 1000 and 1999. The first line of the board accounted for the solution of the exercise. The objective of the exercises was to place five of the fifteen tokens, starting from the top-left cell, in the first line of the board. The remaining ten tokens served as distractors.

The dynamics of the exercise is as follows. The patient is asked to move tokens to the correct location to solve the exercise. Every time the patient moves the wrong token, the therapist (either a human or a robot) will request the patient to move it back to its original location. Eventually, the therapist may provide hints or encouragement (described in the next section). After a number *m* of consecutive mistakes, the therapist will move the correct token on behalf of the patient, as a demonstration. Additionally, if the patient does not perform any move for *n* seconds (timeout), the therapist will intervene, offering additional assistance. As the number of tokens to sort in order to solve the exercise is fixed at five, the number of possible attempts for a patient can be 5*m.

## Developing a fully autonomous robot therapist

The current section describes the development process that helped us to define the main components for deploying a robotic agent that can act in a fully autonomous manner. Specifically, we describe an observational study (see Sect. 4.1), from which we defined the set of cognitive exercises (see Sect. 4.2) for our user studies and that motivated the defined robot’s perception (see Sect. 4.3) as well as its social assistive behaviour (see Sect. 4.4).

### Observational study

Aiming to assess the type of social cues employed by the therapist when administering cognitive exercises to patients affected by cognitive impairment, we ran an observational study. Furthermore, we evaluated, between the set of cognitive exercises described in Sect. 3, those we would have employed later on in our experiment.

In the current study, a therapist was asked to administer a set of cognitive exercises to patients with different degrees of cognitive impairment, offering them assistance and social prompts when he deemed they were necessary. While no bounds were requested on the timing of the assistance, together with the healthcare professionals involved in the project, we restricted the set of therapist’s behaviours to encouragement, hint, and providing the solution.

Eleven patients were invited to participate in the study. Of these, three had mild cognitive impairment, five had mild dementia, and three were affected by moderate dementia (6 males and 5 females, *M* = 72.3, SD = 6.8). According to the guidelines established by the healthcare professionals, the therapist started administering the exercise that was deemed to best fit the cognitive capabilities of the patient, subsequently decreasing or increasing the difficulty depending on their performance (see Sect. 3 for the eight exercises). Furthermore, in order to distinguish the complexity between the three different groups, the mild cognitive impairment group played the exercises with 4 digits, the mild dementia with 3 digits, and the moderate dementia with 2 digits. The number of sessions was not fixed, as it depended on the performance of the patients. The termination criterion was either not being able to solve the exercise or having reached and solved the most difficult one. Each session lasted between 30 and 45 mins, and feedback from the therapist and patients was gathered.

From this study, we found that there were a few key aspects we needed to address before conducting our experiments.

Concerning the exercise, it emerged that it was very important to choose the one that was challenging for the patient; otherwise, no interactions were needed. Regarding the patients, we observed that the type of exercise and their ability to solve it depended not only on their cognitive impairment, but also on their educational level. Some of the participants did not know the difference between even and odd numbers. Some others were not able to solve exercises in which more than one rule was stated. For instance, they could sort tokens in descending order and they could find the odd numbers, but they were not able to sort odd numbers in descending order.

Another interesting behaviour pattern was that the patients got frustrated very easily when they were not able to find the correct token and the therapist needed to reassure them. Additionally, after each exercise, especially in the case where they did not perform well, we needed to explain to them that it was a hard task and making some mistakes was expected. Finally, all the patients, who belonged to the mild and to the moderate dementia groups, very often forgot the objective of the exercise, and thus, the therapist needed to remind them.

With respect to the therapist, we agreed with the healthcare professionals to define seven increasing social assistive levels, that were: (i) turn taking, (ii) encouragement, (iii) reminding task’s rule/s, (vi) suggest row, (v) suggest area, (vi) suggest token, (vii) and offer token. In addition, the therapist’s overall behaviour needed to be always very supportive and their attitude very positive in any situation. Furthermore, from the post-analysis, we found out that when the patients asked about the correctness of the chosen token, after picking one, the therapist provided a sort of feedback, that consisted of a combination of verbal and non-verbal cues, such as nodding his head, changing facial expression, and finally saying words like “Mmmh”, “Ok”, “Are you sure?”, “No”, “Wrong”, etc.

Regarding the therapy sessions, it emerged that the therapist struggled to identify the correct tokens on the board and he experienced difficulty in providing help at the right time, also because of the orientation of the board, which was oriented in the opposite direction with respect to him. Finally, we observed that the therapist, when exposed to more than approximately 2 hours of experiment, started showing signs of fatigue and boredom. On the one hand, administering the exercise to patients affected by cognitive impairment was quite demanding and stressful for him (remembering the solution and deciding in a few milliseconds how to assist them); on the other hand, it was also boring since the task was repetitive.

### Cognitive exercise

From the observational study, the healthcare professionals of Fundació ACE decided to employ three of the eight exercises and set them up as default exercises for each group. The *sort_descending* was chosen for the moderate dementia group, the *sort_descending_odd* for the mild dementia group, and finally the *sort_sum_descending* for the mild cognitive impairment group. For each of them, three different versions were available depending on whether the numbers were of two, three, or four digits. Finally, at any round of the exercise, a sequence of fifteen numbers was defined. Therefore, patients would never play with the same sequence twice.

### Robot’s perceptions

After analysing the relevant states that induced the interactions between the therapist and the patients, we were able to define which inputs were necessary to provide to the social robot in order for it to mimic the therapist’s behaviour.

We decided to restrict the robot’s perceptions to only the contextual information, namely the exercise events. We did not include any information coming from speech and image recognition as these technologies are not reliable enough for being deployed in real-world scenarios and with this target population. We will discuss it in detail in Sect. 9.1.

In order to capture any event triggered by any action on the board, we decided to employ an electronic board (see Fig. 2). The board is based on RFID technology, in which each token is uniquely identified by a unique id (Andriella et al. 2019a). Adopting this board, two main benefits are evident. Firstly, we are guaranteed to have complete and reliable information from the board, regardless of any external condition, such as light, or partial or full occlusion. Secondly, we can capture any information from an event occurring on the board. For instance, we realised from the observational study, that patients quite often sought confirmation after picking a token. Being able to capture that event would give us the possibility to trigger a corresponding robot’s behaviour which replicates that specific therapist’s behaviour (see Sect. 4.4).

**Table 1:** Examples of assistive behaviours implemented in the TIAGo robot

Social category support	Assistive behaviour	Lev	Learnt	Interaction modality	Implementation example
Information Support	Turn taking	0	Yes	V	“It is your turn, please move a token”
	Rules reminder	2	Yes	V	“Remember, you need to sort odd numbers in descending order”
	Pointing a line	3	Yes	V/G	“The correct token is on this line”
	Pointing an area	4	Yes	V/G	“The correct token is around here”
	Pointing a token	5	Yes	V/G	“The correct token is this one”
Emotional Support	Encouragement	1	Yes	V	“Come on, I know you can do it” “Try to pay more attention to the numbers, I believe in you” “Don’t be afraid to make mistakes, move a token!”
Tangible Support	Offering a token	6	Yes	V/G	“The token to move is this one, take it and move it in the correct location”
	Moving a token		No	V/G	“You’ve reached the maximum number of attempts, but don’t worry, I will move the correct token”
	Alerting caregiver		No	V/G	“I don’t know what to do Let’s ask Joan if he can help us”
Appraisal Support	Congratulation		No	V/G/F	“That was a great move!”
	Validation		No	V/G/F	“Congratulations”. “You’re playing as expected!”
	Reassurance		No	V/G/F	“Don’t worry that’s not an easy task, I’m sure you will get better”
	SOCIABLE		No	V/G/F	“Mmmh”, “No”, “Are you sure?”, “Yes”, “Correct”, “Awesome”

The column *Social category support* defines the category according to Cutrona and Suhr (1992). The columns *Assistive behaviour* and Lev specify the different levels of assistance deployed into the robot. The column *Learnt* delimits which assistance has been learnt using the CARESSER Framework. Finally, the column *Interaction Modality* defines the interaction modality employed by the robot to perform an assistive action (V = Verbal, G = Gesture, F = Facial expression)

### Robot’s social assistive behaviour

The next stage in developing a fully autonomous robot is to define its social behaviour. The objective of the robot is not only to actively provide personalised assistance to the patients during the exercise and intervene during an unexpected event on the board, but also to interact socially, improving their motivation and engagement, especially when they are experiencing a hard time, as revealed by the observational study (Winkle et al. 2018). Therefore, in order to correlate the robot’s behaviour with that of the therapist, we implemented a sort of pro-social behaviour, which is defined by Eisenberg and Paul Henry (1989) as “a behaviour which intends to benefit a peer by means of helping, sharing, and comforting.” Pro-social behaviour has been shown to be effective in social relationships and it has been implemented in a social robot by Leite et al. (2014). In their work, Leite et al. employed the social support categorisation system defined by Cutrona and Suhr (1992), which involves five categories: (i) informational, (ii) emotional, (iii) appraisal, and (iv) social network support, and (v) tangible support. Inspired by this work, we decided to reshape the therapist’s assistance according to Leite’s work. Hence, we split up the actions of the therapist during the observational study into four categories, as reported in Table 1. Information support (advice or feedback) includes assistive actions such as reminding of the rules of the exercise (added after the observational study), indicating a line, an area or a token on the board. Emotional support (caring, concern, empathy) includes encouraging actions. Tangible support (concrete assistance) includes the therapist’s actions of offering the correct token to the patient and moving the correct token on their behalf. Finally, appraisal support (compliment and validation) includes all the actions performed by the therapist to praise or reassure the patient after a correct or wrong move, respectively.

As it is shown in Table 1, all these actions were performed using the robot’s voice (verbal cues) and some of them required the robot to move its arm (non-verbal cues). The actions that involve only speech are indicated with the letter “V” under the column Interaction Modality in Table 1. In *turn-taking* action, the robot could decide to only remind the patient that is time to move a token and observe them playing. In *encouragement* action, it can try to motivate the patients to perform a move (e.g., “Come on! I know you can do it”). Finally, in *rules reminder* action, the robot can remind the rules of the exercise (e.g., “Remember, you need to sort tokens in descending order”). Concerning the assistance levels that combine verbal and non-verbal social cues, they are indicated with letters “V/G” under the column Interaction Modality in Table 1. In *pointing a line* action, the robot tells the patient in which line the correct token is located and slides its finger on it (e.g., “The correct token is on this line”). In *pointing an area* action, the robot tells the patient in which area of the board the correct token is located and points its finger in that direction (e.g., “The correct token could be one of those: 32, 55, 12”). Similarly, in *pointing a token* action, the robot tells the patient which is the correct token to move and points its finger to it (e.g., “the correct token is: 55”). Finally, in *offering token* action, the robot tells the patient that it will offer them the correct token to move and therefore picks the token and offers it to the patient on the left or right side of the board (e.g., “The token to move is this one .... take it and move it in the correct location”). If the patient is struggling and cannot find the correct token after a predefined number of mistakes, the robot could decide to intervene and pick and place the correct token (*moving a token* action) in its location (e.g., “You’ve reached the maximum number of attempts, but don’t worry, I will move the correct token”). This is very important, not only to avoid the patient becoming frustrated but also to avoid them getting stuck. This action, as well as the action of offering a token, is implemented robustly thanks to a magnetic gripper.

**Fig. 3:**
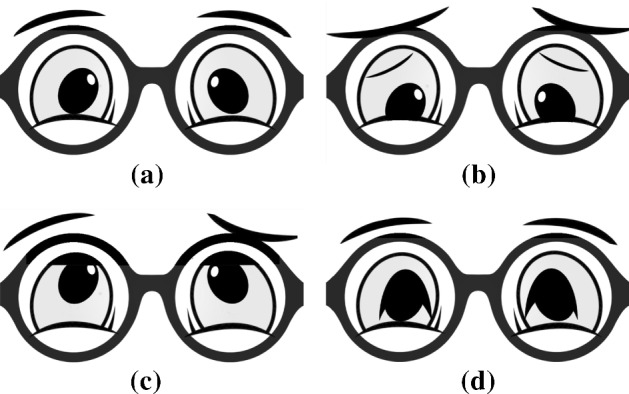
TIAGo’s facial expressions. **a** Neutral, **b** sad, **c** confused and **d** happy

With respect to the appraisal support, the robot can provide support at two different times: immediately after the patient picks a token, and after the patient places a token.

In order to address the need for a quick and effective confirmation after picking a token, we use SOCial ImmediAcy BackchanneL cuE (SOCIABLE) (Andriella et al. 2020a). SOCIABLE is an instantaneous response using a combination of verbal and non-verbal social cues, such as non-word verbal expressions (like “Awesome” if it is the correct token, or “Mmmh, no” if it is the wrong token), facial expressions (see Fig. 3) and head nods. The facial expressions were validated in a pre-study with older adults and healthcare professionals.

Regarding the support after placing a token, the robot was able to use longer sentences (see Congratulation/Validation/Reassurance in the Appraisal Support column of Table 1) also in combination with facial expressions and nodding head actions. This type of action is triggered after every move, and thus, there is no need to include them in CARESSER (see Learnt column in Table 1).

Finally, as we chose to evaluate the robot in a real-world scenario, we developed the robot’s action *Alerting caregiver*, that alerted the therapist when something unexpected happened on the board and the robot was not capable of restoring it by itself. In Table 1, for instance, the robot asks the intervention of Joan, the therapist, to fix the issue.

**Fig. 4:**
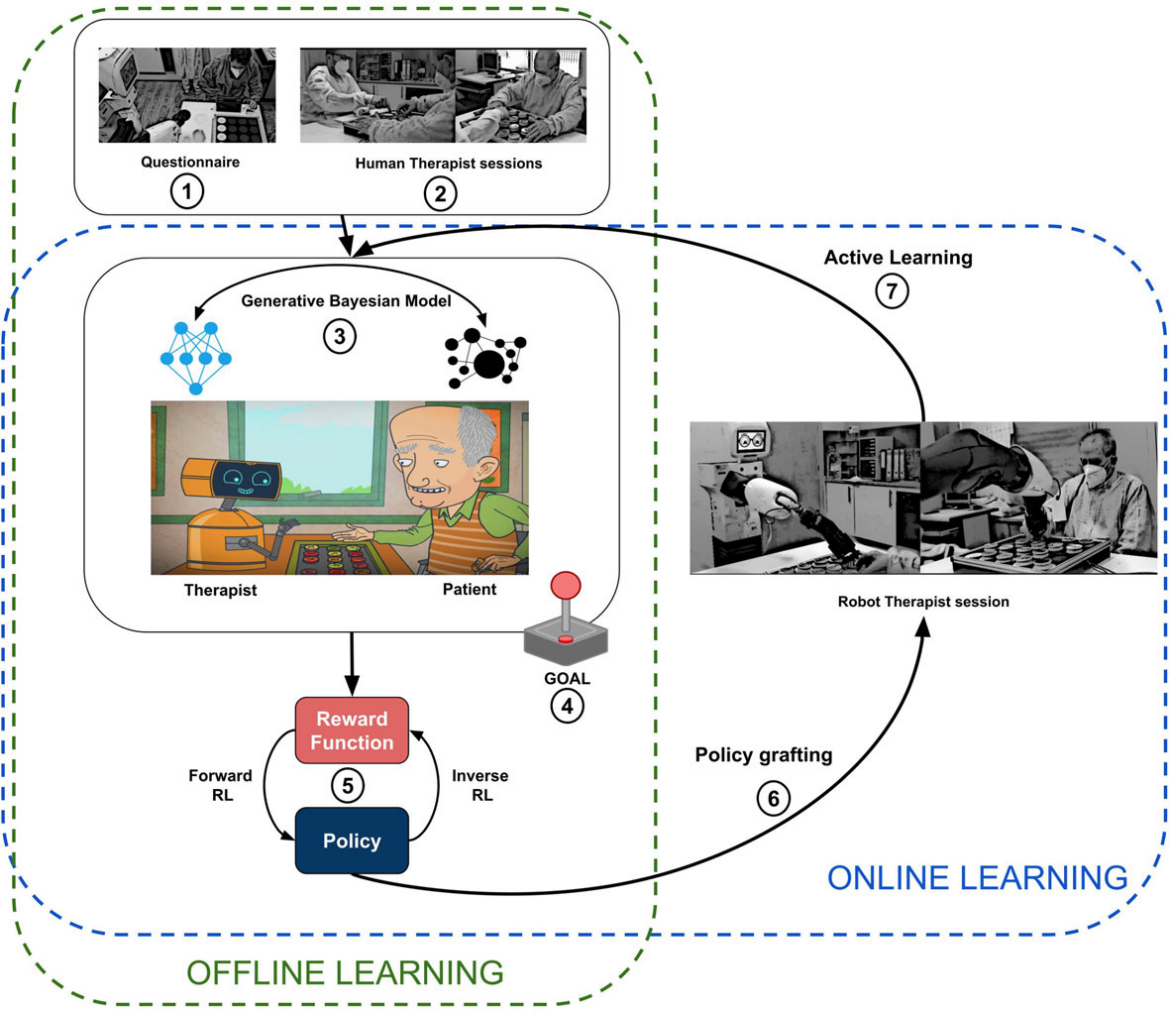
The figure shows the main stages of the CARESSER Framework. In the offline learning phase, firstly, we gather the therapist’s expertise and demonstrations over several sessions (1–2). Secondly, we build the generative Bayesian models of the patient and the therapist (human or robotic) (3), and we run a simulation using the GOAL simulator (4). Thirdly, with the collected episodes output from GOAL, we compute the reward function by means of Max Causal Entropy Inverse Reinforcement Learning and therefore the policy obtained by using value interaction (5). Fourthly, we embed the policy on the robot (6). Then, in the online learning phase, the robot with the initial learnt policy starts administering the exercise to the patient (7). After each session, the generative models are updated (3) with the new data, new episodes are generated (4) and a new reward function and policy are learnt (5–6) and employed in the next session (7)

## aCtive leARning agEnt aSsiStive bEhaviouR framework

The aCtive leARning agEnt aSsiStive bEhaviouR (CARESSER) framework is shown in Fig. 4. CARESSER aims at actively learning social assistive behaviour in order to offer tailored assistance to patients from limited and short-term interactions. This is accomplished by employing a hybrid approach that combines two different methodologies: data-driven and knowledge-driven. We profit from the data-driven method by gathering real data from the interactions between the therapist and the patients during their daily cognitive therapy. On the other hand, we also benefit from the knowledge-driven method by collecting the therapist’s knowledge of the patients’ cognitive abilities by means of a survey. This stage is very important as it would offer the therapist the opportunity to tailor the robot’s behaviour on the basis of the patient’s individual needs. It should be noted that the therapist can be either human or robotic, and we will specify it when it might not be clear.

CARESSER consists of three main components: the generative model, the simulator, and the learning component.

The generative model component builds two probabilistic models (see Sect. 5.1): one of the patients and another of the therapist from the collected data. This is ensured by encoding in the form of probabilities the domain therapist’s knowledge (see Sect. 5.1.2) and the gathering of data (see Sect. 5.1.1) from the interaction between the patient, and either the human therapist or the robot therapist.

The Generative mOdel Agent simuLation (GOAL) component generates episodes, by simulating interactions between the therapist and the patient according to their respective generative models in the cognitive exercise task. In particular, the therapist can provide the patient with different levels of assistance during the session in order for the latter to complete the task (see Sect. 5.2).

The Learning component consists of a Maximum Causal Entropy (MCE) IRL algorithm, that is fed with the simulated episodes and produces the corresponding reward function *R*(*S*, *A*), from which we eventually estimate the patient-specific policy $\pi (s,a)$ using a value iteration algorithm (see Sect. 5.3).

CARESSER consists of two main phases: the offline phase, in which the initial robot’s policy is learnt uniquely by observing the human therapist’s demonstrations and gathering his expertise; and the online phase, in which the robot therapist actively learns to tailor its policy further by interacting with the patients (see Fig. 4).

Specifically, the main steps concerning the offline learning phase are as follows:

- Step 1. Collecting therapist’s knowledge of the patient’s cognitive abilities by means of a survey.
- Step 2. Gathering data from therapist interacting with a patient in an assistive task, in our case the cognitive exercise.
- Step 3. Building two generative Bayesian models: one for the therapist and the other for the patient.
- Step 4. Generating episode by means of GOAL.
- Step 5. Computing the patient-specific policy starting from the estimated reward generated by MCE.
- Step 6. Embedding the learnt policy on the robot.

On the other hand, the steps regarding the online learning phase are as follows:

- Step 7. Playing a session of cognitive exercise between the robot and the patient.
- Step 3. Updating the generative models with the collected data from the current session, generating interactions that take into account the average performance of the patient during the sessions with the therapist and their current performance with the robot.
- Step 4. Generating episode by means of GOAL.
- Step 5. Computing the patient-specific policy starting from the estimated reward generated by MCE.
- Step 6. Embedding the learnt policy on the robot and GO TO Step 7.

**Fig. 5:**
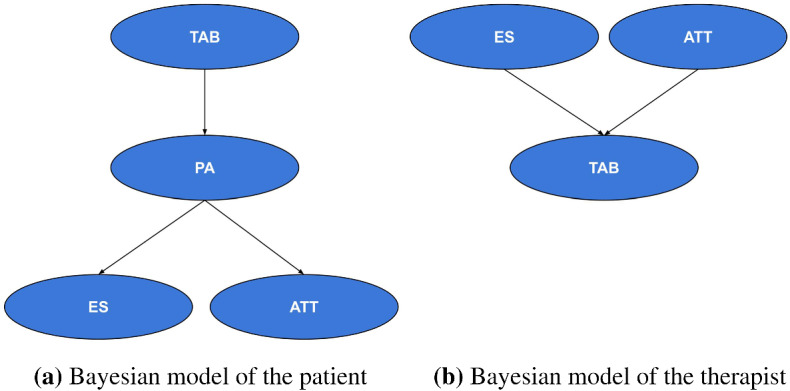
Generative Bayesian models of the patient **(a)** and the therapist (human or robotic) **(b)**. Note that TAB stands for Therapist Assistive Behaviour, PA for Patient Action, ES for Exercise State, and ATT for Attempt

The key aspect of the framework is that it actively improves its qualitative estimation of the patient’s cognitive capability over time and the robot’s assistive actions, updating its current belief after each session between the robot and the patient (Step 3 in online learning phase). In such a way, the robot maintains constant knowledge of the patient’s capabilities; an essential requirement for providing them with tailored assistance, especially in case their performance improves or deteriorates during the therapy.
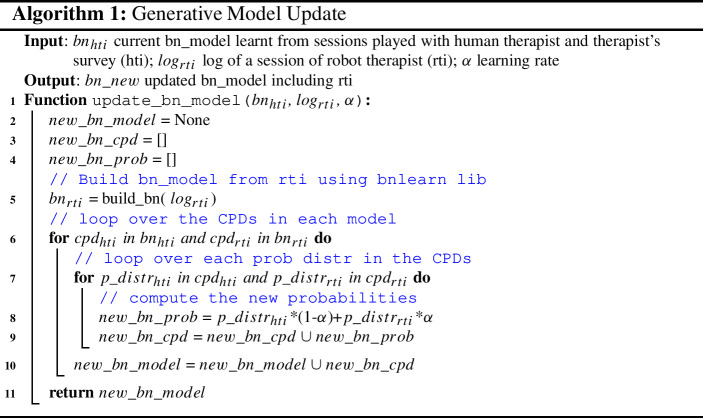


### Generative Bayesian model component

Bayesian Networks (BNs) have emerged as a powerful technique for decision-making under uncertainty. They provide a natural way to handle missing data, they allow the combination of data with domain knowledge (data-driven and knowledge-driven), they facilitate learning about causal relationships between variables, and they can show good prediction accuracy even with rather small sample sizes. In our scenario, we used BNs to build generative models of the patient’s cognitive capability as well as of the therapist’s assistive behaviour. Nonetheless, other techniques for decision-making under uncertainty would also be a feasible option for representing the user and the robot’s cognitive model. This is ensured by using the data collected from the therapist’s demonstrations and their expertise to initialise the models. Furthermore, the data gathered from the robot therapist interacting with patients will be used to actively update the models during the sessions. In this way, we can generate samples that fit the probability distributions for a given patient.

BN is used to model the joint probability distribution over a set of random variables. It is represented as a directed acyclic graph $G=(V,E)$, in which the nodes, $V=\{x_{0},x_{1}, ..., x_{n} \}$, correspond to variables, and arcs, *E*, correspond to probabilistic dependencies between connected nodes. The joint distribution for a BN is equal to the product of *P*(*node*|*parents*(*node*)) for all nodes, as stated below:

$$\begin{aligned} P(X_{0}=x_{0},..., X_{n}=x_{n}) = \prod _{i=0}^{n}P(X_{i}|\hbox {Parents}(X_{i})) \end{aligned}$$

**Table 2:** Variables and respective values of the two BNs

Variable	Value						
PA	correct_fast	wrong_fast	correct_medium	wrong_medium	correct_slow	wrong_slow	timeout
	(t<timeout/3)	(t<timeout/3)	(timeout/3<t<2*timeout/3)	(timeout/3<t<2*timeout/3)	(2*timeout/3<t<timeout)	(2*timeout/3<t<timeout)	t>timeout
TAB	lev_0	lev_1	lev_2	lev_3	lev_4	lev_5	lev_6
ES	beginning	middle	end				
ATT	att_1	att_2	att_3	att_4			

Figure 5 shows the BNs used to represent the patient and the therapist. The two BNs define the state-space variables and the relation between them. Four variables are defined: PA (Patient Action), TAB (Therapist Assistive Behaviour), ES (Exercise State), and ATT (Attempt). The possible values for each variable are shown in Table 2.

In Fig. 5a, we show the BN depicting the patient. The joint probability function is:

1 $$\begin{aligned} \hbox {Pr(PA, TAB, ES, ATT)}=\hbox {Pr(TAB) * Pr(PA|TAB) * Pr(ATT|PA) * Pr(ES|PA)}, \end{aligned}$$

and represents a distribution over the state space. In our scenario, we are interested in inferring the probability of PA, using variable elimination algorithm, given some evidences ES, TAB and ATT.

With respect to the BN of the therapist (see Fig. 5b), we are interested in inferring the probability of TAB given ES and ATT. As can be noted, the BN of the therapist is very simple and consists of a network with a single effect (TAB) for multiple causes (ES and ATT). Simply put, the probability of TAB is directly accessible from the conditional probability distribution (CPD) table and there is no need to compute it. The main reason for creating two different models is that with these we aim to capture a temporal dependency between the variables. That is, in the patient’s model, the therapist’s behaviour causes a patient’s movement, which in turn causes a change in the state (exercise and attempt), while in the therapist’s model, the current state (exercise and attempt) prompts the therapist’s assistive action.

Once initialised, the BNs contain the patient’s cognitive abilities on the specific task and the therapist’s preferred levels of assistance. Differently from many studies that employed static BNs, here we actively update their CDPs, when an exercise session between the robot and the patient is concluded. This can be accomplished by acquiring the new samples and normalising them with respect to the current probabilities. In this manner, session after session, we have a more reliable model of the therapist and the patient.

Algorithm 1 shows the steps taken on by the system to update its BNs. Bear in mind that this can happen only after at least one session played by the patient with the robot. Firstly, a BN is built from the data collected in the interaction between the robot and the patient (line 5). This is done by collecting how many times events of interest occurred (TAB, PA, ES, ATT) and then representing them in the form of probabilities. Secondly, we build a new BN, which normalises the probabilities of each CDP according to the following updating rule (lines 6–9): $(1-\alpha )*\hbox {Pr}(X_{t-1})+\alpha *\hbox {Pr}(X_{t})$, where $\hbox {Pr}(X_{t-1})$ is the current probability of X and $\hbox {Pr}(X_{t})$ is the new probability of *X*, where *X* can be any of the variables defined above. In this work, we set $\alpha =0.6$.

Finally, for computing the inference of the two BNs, we used bnlearn^1^ a library available in different programming languages, including Python and R.

#### Encoding therapist’s demonstrations

In order to initialise the two generative models discussed above, we firstly gathered data from the human therapist interacting with patients in a user study (see Sect. 7). Although the exercises were customised for the experiment described in this article, cognitive sessions are part of the daily activities for patients attending the centre. Therefore, collecting data from their interactions is a reasonable procedure.

After each session, between the therapist and the patient, a log is saved with the information regarding the interactions. Consequently, the two BNs are filled with the data collected. This is accomplished by counting the occurrences of an event and then normalising it with respect to its probability distribution. Although the variables PA, ES, and ATT are recorded automatically during the interactions thanks to the electronic board, the assistance of the therapist needs to be labelled. Therefore, with a simple GUI, the experimenter is able to label the therapist’s behaviour according to the actions defined in Sect. 4.4. In this way, we can assess TAB, which is required for the generative model of both the therapist and the patient.

**Fig. 6:**
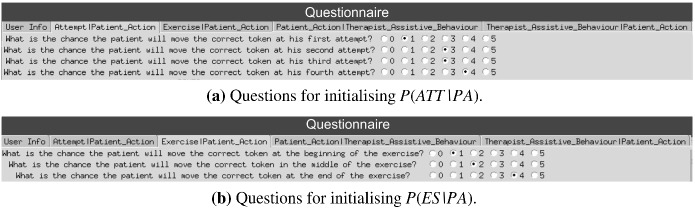
Example of questions administered to the therapist

#### Encoding therapist’s knowledge

Apart from the data collected from the therapist’s demonstrations, to initialise the BNs, a kind of questionnaire is administered to the therapist before the patient performs the exercise. It surveys the therapist on the patient-specific cognitive ability for the proposed task. This stage is very important as it offers the therapist, through a GUI, the opportunity to set up not only the expected patient’s actions but also the assistance the therapist would provide them. In Fig. 6, we show an example of questions asked by the therapist. Each question is formulated in natural language and the therapist is requested to provide a score between 0 (that event is highly unlikely to happen) and 5 (that event is very likely to happen) of the likelihood that an event may occur. It is important to note that the score employed to ease the therapist’s understanding refers to probabilities normalised between 0 and 1, in which 0 corresponds to 0.0 and 5 to 1.0.

**Table 3:** Initialisation of the variables *Pr(ATT|PA)* (a) and *Pr(ES|PA)* (b) according to the questions of Fig. 6 (in bold). Note, that the remaining probabilities for variables *wrong* and *timeout* are estimated starting from the variable *correct*. Note also that we do not get information on the patient’s reaction time from the questionnaire (slow, medium, fast)

(a) Initialisation of *Pr(ATT|PA)*		
ATT	PA	Pr
**att**_**1**	**correct**_{**slow**, **medium**, **fast**}	**0.2**
**att**_**2**	**correct**_{**slow**, **medium**, **fast**}	**0.6**
**att**_**3**	**correct**_{**slow**, **medium**, **fast**}	**0.6**
**att**_**4**	**correct**_{**slow**, **medium**, **fast**}	**0.8**
att_1	wrong_{slow, medium, fast}	0.4
att_2	wrong_{slow, medium, fast}	0.2
att_3	wrong_{slow, medium, fast}	0.2
att_4	wrong_{slow, medium, fast}	0.1
att_1	timeout	0.4
att_2	timeout	0.3
att_3	timeout	0.2
att_4	timeout	0.1

(b) Initialisation of *Pr(ES|PA)*		
ES	PA	Pr
**beg**	**correct**_{**slow**, **medium**, **fast**}	0.2
**mid**	**correct**_{**slow**, **medium**, **fast**}	0.4
**end**	**correct**_{**slow**, **medium**, **fast**}	0.8
beg	wrong_{slow, medium, fast}	0.4
mid	wrong_{slow, medium, fast}	0.3
end	wrong_{slow, medium, fast}	0.1
beg	timeout	0.4
mid	timeout	0.3
end	timeout	0.1

By way of illustration, we show for the questions reported in Fig. 6 the process of instantiating the variables $\hbox {Pr(ATT|PA)}$ and $\hbox {Pr(ES|PA)}$ (see Table 3). As shown in Fig. 6, we limit them to the only correct action of the patient (highlighted in bold in Table 3). Therefore, for the patient’s wrong move and timeout, the likelihoods are estimated by simply equally distributing the remaining probabilities between them. It is also important to mention that the reaction time of the patients is not set by the therapist; therefore, at the beginning, there is no difference with respect to the fast, medium, and slow options in terms of probabilities, and they are all initialised at the same values (see Table 3).

Despite the fact that the collected knowledge is not very accurate, this step is very important to learn a coarse prior over the entire states space, as it complements the information about states not visited during the interaction with the therapist. Furthermore, this has the additional benefit of driving the learning process towards states that are more suitable for a specific patient (personalisation) and therefore reducing the selection of inappropriate assistive actions.
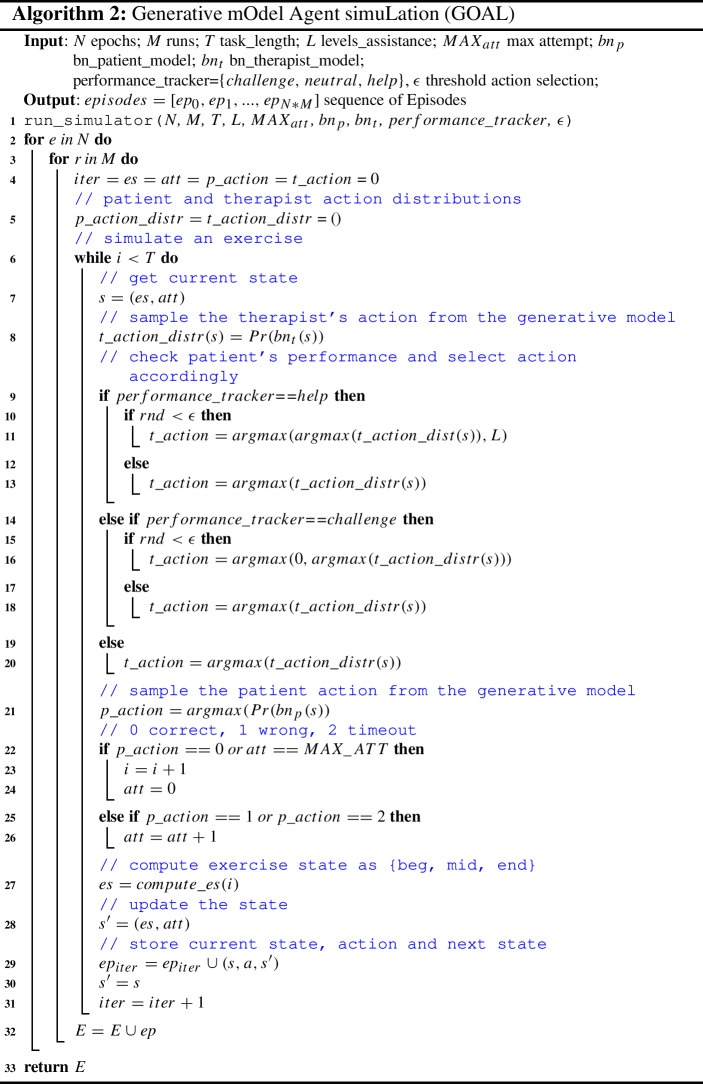


### Patient-specific simulator component

Generative mOdel Agent simuLation (GOAL) is accountable for generating a sequence of episodes by simulating the interactions between two agents in a sequential assistive task: the therapist agent that knows the solution of the task and therefore can provide assistance and the patient agent, that, on the contrary, has some skills but not all of them, to solve the exercise. GOAL is an evolution of a previous simulator called Persona-behaviour simulator (Andriella et al. 2019b). In Andriella et al. (2019b), the simulator acquired information from the therapists according to four dimensions (hearing, memory, attention and reactivity) and attempted to generate with a high-level abstraction, different patients’ profiles. Differently from Andriella et al. (2019b), GOAL is fed with real data and not with fictitious data modelled according to a given distribution. Finally, GOAL generates interactions that are specific and unique for a given patient and not for a generic patient’s profile.

The main steps of GOAL in the cognitive exercise task defined in Sect. 4.2 are shown in Alg. 2. Firstly, we need to initialise *N* and *M* which are the number of epochs and runs, respectively. Next, we configure the number of levels of assistance the therapist will offer to the patient agent and the two generative Bayesian models of the therapist and the patient (Sect. 5.1). Finally, it requests to set the $performance\_tracker$ variable, which defines whether the therapist’s behaviour needs to be reshaped as the performance of the patient has changed. This is a feature of the simulator that is key to achieving the desired objective of a robot therapist capable of keeping the patients effectively challenged. Indeed, as explained in Sect. 5.1, with the acquired information we are able to initialise a generative model of the therapist and of the patient and therefore to run the simulation. Nonetheless, this approach, as it is, would not take into account the fact that the patient’s capabilities can change over time. That can occur for several reasons: for instance, the patient’s attitude toward the task, the robot’s novelty effect, or the task’s learning effect. Hence, the simulator, depending on that, should be able to generate a therapist’s behaviour that reflects the patient’s changes. This is achieved by relaxing (challenging) or increasing (helping) the assistance to the patient during the simulation. To do so, the therapist needs to evaluate the patient’s performance. In our case, the average score of the patient $m_{s}$ is collected during the interaction of the human therapist with the patients. Then, if $c_{s}$ is the current score of the patient during the simulation, we check if $|m_{s}-c_{s}|$ is bigger than a threshold *thr*, if so the simulator has to change its behaviour to reshape the therapist’s policy. This is done by checking whether this difference is positive (challenging, as it is performing better) or negative (helping, as it is performing worse).

After having initialised these variables, we can run the simulation which will iterate $N*M$ times. During each simulation (lines 6–31), the therapist helps the patient to perform the correct action in a given state. Specifically, we get the current state (line 7) and sample the therapist’s action from its generative Bayesian model (line 8), that is a probability distribution over its actions ${ass}\_{lev}$ = $\hbox {Pr(lev}\_0, ..., \hbox {lev}\_6)$, where the levels are those defined in Sect. 4.4. Next, we check the value of $performance\_tracker$ (lines 9–20). Depending on it, the therapist will sample the action with the highest probability, $lev\_x$, from the current generative Bayesian model or reshape its policy. In particular, if $performance\_tracker$ is equal to *help* (line 9), the therapist will sample its action of assistance from those that are more assistive with a probability $\epsilon$ (line 10). In contrast, if $performance\_tracker$ is equal to *challenge* (line 11), then the therapist will select its action of assistance from those that are less assistive with a probability $\epsilon$ (line 12). Otherwise, we select the action of assistance with the highest probability (line 20). Then, we sample the patient’s action from its generative model (line 21); depending on it, we update the state of the task. If the action is correct or the patient reaches the maximum number of attempts (line 22) on a given token (the therapist will perform the correct move on its behalf), the task progresses to the next step (lines 23–24). On the contrary, if the action of the patient is incorrect or it reaches the timeout (line 25), we only increase the attempt counter (26). Finally, we update the exercise state, store the $state-action-next\_state$ triplet and compute the next state of the task (lines 27–31). When the task is completed, the episode is saved in the list of Episodes *E* (line 32).

In order to validate that the new policy, selected according to the logic described above (lines 9–20) satisfies our desired behaviour, GOAL runs until the average simulated patient’s performance does not achieve the expected one for at least *n* episodes. This means that if this minimum number of episodes is not reached, GOAL would ask to rerun the simulation with a different $\epsilon$ value.

### Learning component

The main goal of CARESSER is to find the robot’s socially assistive policy that is the most appropriate to the patient’s individual needs. Most of the approaches envisage addressing this goal by designing a reward function that captures the desired behaviour and then employing a forward reinforcement learning algorithm to learn the corresponding policy (see Sect. A.2). Defining a reward function might be time-consuming and very often quite complex as a lot of aspects need to be considered. Furthermore, in the scenario we are proposing, the reward is highly dependent on the patient and the therapist. From the patients, because the reward must be defined to be tailored to them, as each patient has their own needs. From the therapist, because the design of the reward might depend on their own belief and manner of leading the therapy. Therefore, in this article, we propose an alternative approach to achieve the aforementioned goal based on IRL. In IRL, the task is to take a set of expert’s demonstrations and extract from these, an approximation of the expert’s reward function for the given task. Differently from a policy, a reward captures the essence of the task as it quantifies the quality of certain actions in a given state. In our scenario, the expert is the therapist, who can be either a human or a robot, and the demonstrations are therapist’s social assistive behaviours. The therapist is requested to assist the patient while they are playing a cognitive exercise. The goal is achieved when the patient places the tokens in the correct order.

Firstly, we formalise the task as an MDP = $\langle S,A,T \rangle$ /*R* (see Sect. A.1).

The state space (*S*): consists of the following variables: $\hbox {ES}=\{beg, mid, end\}$ is the exercise state, $\hbox {ATT}=\{att1, att2, ..., att4\}$ is the attempt of the patient in a given state ES, and $\hbox {PA}_{t-1}=\{correct, wrong, timeout\}$ is the action performed by the patient in the previous state. In total, the state space consists of $|\hbox {ES}|\times |\hbox {ATT}|\times |\hbox {PA}|$=36 states.

The action space (*A*): is discrete and the therapist has 7 available actions: $\hbox {TAB}=\{\hbox {lev}\_0, \hbox {lev}\_1, ..., \hbox {lev}\_6\}$. Each action corresponds to the assistance offered by the therapist as described in Sect. 4.4.

The transition probabilities ($T(S', A, S)$): define the model of the environment and they are initialised with the data collected during the therapist interacting with the patients (see Sect. 5.1.1) and updated during the interactions between the robot and the patient. Note that although the proposed algorithm needs a model of the environment, there are others that do not require the dynamics of the environment.

Secondly, we propose to solve the problem of finding the therapist’s assistive actions that best fit the patient by employing the Maximum Causal Entropy algorithm (see “Appendix A.3”). The problem is reduced to find a reward function *R*, starting from the feature vector $\pi _{i}(s)$ and the therapist’s demonstrations *E*.

The feature vector ($\phi _{i}(s)$): describes behaviour in terms of the state of the exercise, the therapist’s assistance and the patient’s actions. We encode into the vector, the distance to the goal, $D_\mathrm{{goal}}$, the number of total attempts $N_\mathrm{{attempt}}$, the assistance offered by the therapist, $A_\mathrm{{therapist}}$, the action of the patient, $A_\mathrm{{patient}}$, and the reaction time of the patient, $\hbox {RT}_\mathrm{{patient}}$.

The therapist’s demonstrations (*E*): are collected by running GOAL which generates episodes according to the patient and therapist’s generative models. They are described in terms of $(s, a, s')$, where *s* is the current state, *a* is the action performed by the therapist and *s’* is the next state.

The goal of the algorithm is to optimise a reward function to generate a policy $\pi$ with a feature expectation vector ${\mathbf {f}}_{\pi }$ that satisfies ${\mathbf {f}}_{\pi }$=${\mathbf {f}}_{E}$, where ${\mathbf {f}}_{E}$ is the feature expectation vector estimated using the set of demonstrations *E*. Specifically, the gradient descent process of MCE is in charge of adjusting the reward function and minimising the difference between the two feature expectation vectors ${\mathbf {f}}_{\pi }$ and ${\mathbf {f}}_{E}$ (see Sect. A.3). We assume that the generation of a similar dynamic situation in a task implies similar behaviour.

#### The framework

The main stages of the CARESSER framework are summarised in Alg. 3.

CARESSER takes as input the parameters to set up: GOAL, the MDP, the MCE algorithm, and the value interaction algorithm.

The framework is queried at the beginning of each session between the robot therapist and the patient. Note that the sessions between the human therapist and the patient are only used to initialise the system, that is, to learn the policy the robot therapist adopts during the first session. Therefore, CARESSER firstly checks whether it is the first session (line 2). If not so, it gets the data collected from the previous session and updates the two generative models (lines 3–4). Then, it employs GOAL to generate a sequence of demonstrations *E* (line 5). Next, the demonstrations are used to initialise the MDP (line 6) and execute the MCE algorithm (line 7) as defined in Sect. A.1. The algorithm returns the reward function *R*, which is then employed to recover the robot’s policy $\pi$ (line 8) using a value iteration algorithm. The code repository of the whole framework is available here.^2^
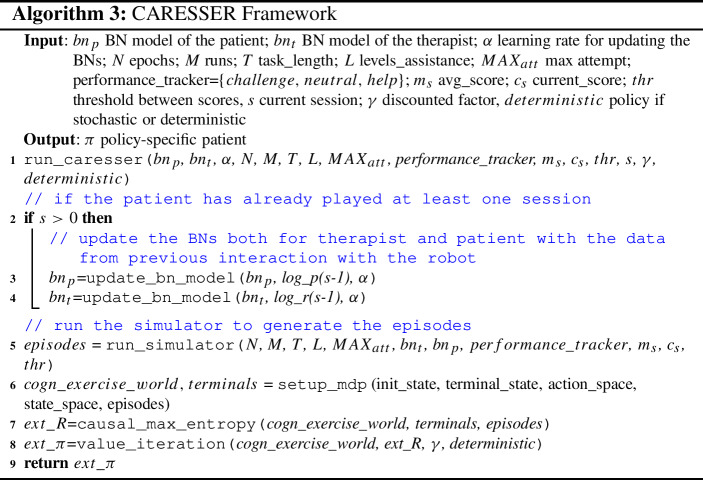


## Methods and materials

In this section, we provide the methods and procedures used in the two user studies, namely the study with the human therapist (see Sect. 7) and the study with the robot therapist (see Sect. 8), which will be described in detail in the next sections.

The studies were set up as a within-subject design, in which the same patient interacted both with the human therapist and the robot therapist during six sessions. It is important to note that a learning effect might be present as we could not randomise the order in which the two user studies were carried out. Nonetheless, we argue that this was very unlikely to happen for the following reasons: (i) the patients were used to playing this kind of exercise with the therapists during their daily therapies, (ii) immediate and long-term free recall deficits are common in patients with cognitive impairment (Carlesimo and Oscar-Berman 1992; Andrés et al. 2019) and the time between the sessions with the therapist and the robot was long enough to assume they could not remember them, and (iii) the exercise has been designed with numbers, which are, in general, more difficult to remember (Hulme et al. 1991).

To demonstrate the presence or absence of an effect, we analysed the data using simple or multiple regression analysis. Using regression analysis can help consistency in comparing or replicating results across different studies and is also a very convenient method to check for confounding variables (Hoffman and Zhao 2020). Additionally, to evaluate the presence of an effect between sessions, we used Friedman’s (Friedman 1937) as omnibus test and Conover’s post-hoc multiple comparison tests (Conover 1998) to discern which of the pairs had significant differences.

### Hypotheses

We evaluated the following hypotheses:

**H1**: Using CARESSER, the assistance offered by the robot is deemed acceptable more often by the therapist, session after session, eventually converging to the desired policy at the end of the sixth session.
**H2**: The perceived cognitive demand required by the patients while playing with the robot is not significantly different than when they play with the therapist.
**H3**: The performance of the patients while playing with the robot is significantly different than when they play with the therapist.
**H4**: The estimation of the patients’ performance using the simulator is not significantly different than the patients’ performance when they play with the robot.
**H5**: Using CARESSER, the robot manages to keep constant the patients’ performance over the sessions better than the therapist.

With H1, we aim at addressing RQ1a and validating the effectiveness of the assistance offered by the robot. On the other hand, the experimental hypotheses H2 and H5 provide arguments to tackle RQ1c. We aim that the robot can provide tailored assistance better than the therapist (H5) without too much effect on the patients’ mental workload (H2). Finally, both hypotheses H3 and H4 help to shed some light on RQ1b. While human therapists are considered the gold standard and the upper bound of a robot-assisted intervention, in our use-case, given the preliminary results from the observational study, we hypothesised that the robot would have a different impact on the patient’s performance because of (H3): (i) the eventual boredom and fatigue experienced by the therapist during the therapy, which could reduce their effectiveness, and (ii) its presence that would have a positive effect on the attention and focus of the patients, as reported by Pino et al. (2020). Finally, we hypothesise that the GOAL simulator would be able to accurately approximate the patient’s performance and the therapist’s assistance by means of their generative models (H4).

**Fig. 7:**
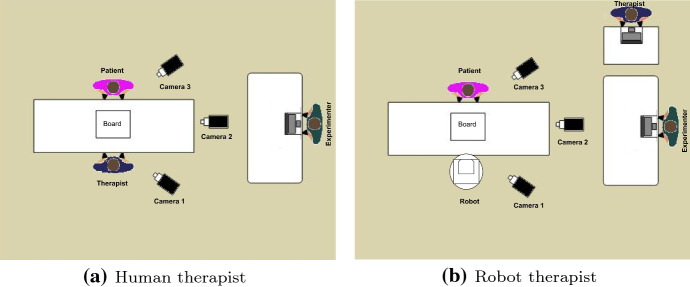
User studies set-up

### Experimental setting

In order to foster natural interaction between the patient and the therapist (human or robotic) and, more importantly, to make the patient comfortable, we decided to carry out in situ user studies, whereby the experiment was conducted in the rooms where patients are used to attending cognitive psycho-stimulation workshops and occupational therapy classes. Figure 7 shows the experimental set-up for the human therapist study (see Fig. 7a) and the robot therapist study (see Fig. 7b), respectively. The therapist was seated in front of the patient, and the board was placed on the table. The exercise area was semi-closed to avoid any source of distraction for the patient. Apart from the therapist and the patient, the experimenter was also present in the room. The experimenter was seated on a different table (2 m away) and was in charge of initialising the session and ensuring that the system worked as expected. In the robot therapist study, the human therapist was still present during the experiment to help when needed and to assess the quality of the robot’s assistive actions. The therapist was sitting behind the patient in order to not interfere with the experiment.

Three cameras were installed to record audiovisual data for further analysis of verbal and non-verbal communication and behaviour of the therapist and patients during the sessions. Two cameras were located at the side of the patient and the therapist (cameras 1 and 3), while a third one was placed above to capture the whole scene (camera 2).

### Inclusion criteria

Patients from Fundació ACE were selected for this experiment using the following inclusion criteria: more than 50 years old; diagnosis of dementia according to McKhann et al. (2011) or mild cognitive impairment according to Petersen et al. (1999); Mini-Mental State Exam (MMSE) score above 18; Global Deterioration Scale (GDS) not higher than 5 and Clinical Dementia Rating Scale (CDR) not higher than 2; willingness to participate in the experiment, and signature of informed consent. We excluded patients who, in the opinion of the investigator, lacked adequate literacy, visual, or auditory acuity to complete the experiments. Additionally, patients with severe apathy, unstable medical conditions, severe neuropsychiatric symptoms, legal incapacity, or inability to complete the protocol were also excluded.

### Study approval

This research study has been approved by the Ethical Committee of the Universitat Internacional de Catalunya (UIC) and revised by the Ethical Committee of the Spanish National Research Council (CSIC).

**Table 4:** (a) Summarises the sample’s characteristics of the participants selected for the study (*N* = 26), while (b) summarises the sample’s characteristics of the participants who completed the study (*N* = 22)

(a)	
Demographic features	*N* = 26
Age (*M* and SD)	74.77 (9.31)
Gender (*n* and %)	
Male	14 (53.9)
Female	12 (46.2)
Years of education (*M* and SD)	10.31(4.06)
MMSE (*M* and SD)	24.46 (3.37)
Stage of disease	
Mild cognitive impairment	11 (42.3)
Mild dementia	15 (57.7)

(b)	
Demographic features	*N* = 22
Age (*M* and SD)	74.43 (9.5)
Gender (*n* and %)	
Male	12 (54.5)
Female	10 (46.5)
Years of education (*M* and SD)	9.62 (3.54)
MMSE (*M* and SD)	24.57 (3.59)
Stage of disease	
Mild cognitive impairment	10 (46.5)
Mild dementia	12 (54.5)

Note that *M* stands for mean and SD for standard deviation

### Participants

Twenty-six patients from Fundació ACE were included in the experiment, 22 of whom completed the two studies (human therapist study and robot therapist study). We had 1 screening failure due to lack of capacity in the investigator’s opinion to complete the protocol and 3 dropouts: 2 of them because of the withdrawal of informed consent and the other patient missed one of the two studies. Table 4a depicts the characteristics of the sample.

### Apparatus

This experiment was based on a set of cognitive exercises designed by the healthcare personnel of Fundació ACE. The exercise was administered by means of an electronic board and tokens with NFC technology (see Sect. 3). Specifically, each token had an iron strip on the top, to which it was possible to attach/remove numbered card-boards.

As a robotic platform, we employed the TIAGo robot. Apart from its versatile capability in terms of movements and manipulation, the TIAGo platform offers several degrees of personalisation. In our TIAGo, the end-effector has been replaced with a magnetic gripper, which eases the manipulation of the tokens, especially for actions such as pick and place. This is the reason we decided to insert an iron strip on the top of each token. Furthermore, we replaced TIAGo’s original head with an LCD 7-inch screen. As a result, we were able to reproduce any facial expression and use it as an additional interaction modality (see Fig. 3).

### Protocol

The experiment was conducted in three different facilities of Fundació ACE: at the Daycare Hospital for Pharmacological Treatment, at the Daycare Centre and Memory Unit, and finally at the Daycare Hospital. The two user studies were carried out over two months. Due to the availability of the patients, the schedule of the therapist, and the restrictions of the pandemic for COVID-19, we were able to perform the experiment only two days per week. Therefore, every two weeks, we could conduct the experiment with 6–7 patients. The first week, we carried out the study with the human therapist, and the week after, the study with the robot therapist.

The experimental protocol was the same for both the user studies, with a few exceptions that will be highlighted in the next few paragraphs.

Each participant was accompanied by one of the caregivers from the centre to the experimental room. The therapist and the experimenter received them and explained the purpose of the study. They were told that they would be part of an experiment that consisted of two phases, and they were asked for their availability to perform the second phase in one week. If the patient agreed to participate, the experimenter asked them to fill in an informed consent form, which included the authorisation to gather data for scientific purposes. Next, the experimenter, with the help of the therapist, explained the kind of exercises. After that, and before starting the experiment, the experimenter conducted a demonstration that consisted of playing one exercise to address any doubts that might arise concerning the rules of the exercise. In the robot therapist study, the demonstration was also important to show the patients the different interaction modalities of the robot and its range of movements. This stage lasted between 5 and 20 min. Afterwards, the experiment started. In the human therapist study, the patient was asked to play a warm-up exercise in order to check whether the level defined by the therapist was the most appropriate. This stage lasted between 5 and 15 min. In the case of the robot therapist study, this stage was skipped as the exercise was already defined in the previous study (human therapist study). Next, participants were asked to play six sessions with the therapist.

After each session, a break of 3–5 min was offered to the patients while the experimenter placed a new sequence of numbers on the board. This is to avoid the patients memorising the tokens. Additionally, the sequences used in each session were randomly chosen using a balanced Latin square to avoid any learning effect. Finally, before the exercise started, the board was covered until the moment the exercise started. After the sixth session, the patients were asked to fill in the NASA TLX test.

All the interaction sessions were video recorded, with the exception of those patients who did not consent to the recording. Additionally, for each patient, the interaction logs were saved. On average, a study with a patient lasted between 1 and 1 h and 20 min. Hence, each patient was involved in the two phases of the experiment for approximately 2 and a half hours. After the six sessions were completed and the test filled in, the experimenter asked the patients about their experience, to gather informal feedback.

### Evaluation measures

To address our hypotheses (see Sect. 6.1), we collected objective and subjective measures on both the studies: human therapist and robot therapist.

Concerning the objective measures, we grouped them into exercise measures and personalisation ranting. The exercise measures described the patients’ performance per session, such as their total number of attempts, mistakes, and timeout that came out from the simulation, from their interaction with the therapist, and from their interaction with the robot. Finally, during the two studies, we also collected participants’ reaction time (time to pick a token after assistance was provided), elapsed time (time to place a picked token in a location), and completion time. These measures will help to tackle H3, H4, and H5. The personalisation rating aimed at characterising the robot therapist’s policy, i.e. the assistive behaviour offered by the robot to the patients. In this regard, the human therapist, during the robot therapist study, was asked to rate the robot’s social behaviour. He provided a rating on a 5-points scale, in which 1 stands for “I strongly disagree”, and 5 stands for “I strongly agree”. He was also requested, in case he disagreed, to note down what he deemed was the correct behaviour. This rating provided by the therapist will deal with H1.

Regarding the subjective measure, we collected data administering the NASA Task Load Index (NASA-TLX) (Hart and Staveland 1988). The NASA-TLX test was used to assess the patients’ perceived workload during the task. With the TLX test, we aimed to estimate the effort patients have to exert both mentally and physically to solve the exercise. In TLX, the workload is modelled along six dimensions: mental, physical, temporal demands, frustration, effort, and performance. One important preliminary stage in TLX is to define the weightings of each dimension. Although originally this is requested of each of the participants, we asked the therapist to define which of these dimensions were the most relevant for the defined task. As in the presented use-case, the physical workload was not so relevant compared to other dimensions, and they decided to assign it a lower weighting. On the other hand, as they believed frustration and mental demand were very important measurements, they assigned them a higher weighting. To summarise, to the temporal demand, effort, and performance dimensions were assigned 0.15, while to frustration and mental demand, 0.25 and finally to physical demand, 0.05. In order to facilitate the evaluation for the patients, we modified the scale originally from low to high to 1 to 10. Hence, the participants could provide a score, based on their perception, between 1 and 10 for each dimension. The TLX will address H2.

## Study with the human therapist to model interactions

The objective of this study is twofold. Firstly, we aim at gathering data from the therapist and the patients that later on will be used by CARESSER to build the generative Bayesian models and produce an initial patient-specific policy to embed in the TIAGo robot.

Secondly, we evaluate the strategies employed by the therapist to keep the patients engaged during the exercise, that is, providing tailored assistance in order to maintain their performance, as much as possible, constant. It is noteworthy that the rationale behind any assistive behaviour, to some extent, depends on the therapist himself. This was the main reason for involving only one therapist in the study, who was also the examiner of the robot’s behaviour in the study presented in Sect. 8. Though it can be argued this is a limitation, it is a very reasonable condition; as generally, therapists have their own way of approaching, interacting, and engaging with patients.

### Therapist’s selection

The therapist was selected from among the healthcare professionals available at the Hospital. We looked for a professional who was used to administering clinical therapy, such as exercises and assessment tests for memory, attention, and language. The selected therapist is a psychologist with expertise in cognitive stimulation therapies for patients at different stages of dementia. He had limited knowledge of robotics and artificial intelligence but a very deep understanding of patients’ cognition. Whereas he was involved in the experimental design (defining the robot’s social behaviour) and participated in the observational study, he was not aware of the hypotheses and the research questions, and therefore, he had no incentive to bias the results to fit them.

### Study introduction

We present a study in which a therapist (see Sect. 7.1) was asked to administer a cognitive exercise to patients suffering from mild dementia and mild cognitive impairment, offering them assistance and hints. The levels of assistance that the therapist could employ were those defined in the observational study (see Sect. 4.1) and are listed in Table 1.

Firstly, the therapist was asked to fill in a questionnaire on the patient’s cognitive ability for the given exercise (see Sect. 5.1.2). Next, the therapist administered the cognitive exercise to the patient. The therapist administered the exercise for six sessions, changing after each round the sequence of numbers while keeping the objective of the exercise always the same (e.g. sorting tokens in ascending order).

The therapist’s demonstrations (see Sect. 5.1.1), as well as the patient’s actions in each session, were recorded. Specifically, a log with the variables of interest for initialising the generative Bayesian models was generated (see Sect. 5.1). This data was then provided to CARESSER, which, according to the offline phase presented in Fig. 4, builds the patient and the therapist’s generative models, runs a simulation by use of GOAL, produces episodes, and learns first a reward function and finally a patient-specific policy (see Alg. 3).

### Example of a session

In the video,^3^ we show an example of a session between the therapist and a patient. As it can be noticed, the therapist assisted the patient every time she made an incorrect move or a timeout was reached. Furthermore, the therapist offered SOCIABLE when the patient picked a token. Regarding the patient’s performance, it is possible to see how the patient was quite fast at finding the correct tokens in the first stages when the exercise was supposed to be harder. On the contrary, the patient struggled to find the last token. This highlights how assumptions regarding assistance based on the stage of the exercise might not stand.

Finally, besides the assistance offered by the therapist, it can be observed how the patient, looking for the fourth token to place, moved a token already placed correctly to the wrong location. This is important to highlight the complexity and unexpected situations that could occur by conducting experiments in the wild with real users and a real board, as those are the kind of events a fully autonomous robot should be able to detect and recover from.

**Table 5:** Results of the NASA TLX test from the human therapist study

	Mental	Physical	Temporal	Effort	Performance	Frustration	Overall
*M*	5.15	1.49	4.26	6.78	5.4	2.61	4.48
SD	1.84	0.5	1.87	1.48	1.63	1.51	0.65

### Results and discussion

In this section, we present and discuss the results of this study.

Regarding the subjective measure, that is, the NASA TLX test, the results are reported in Table 5. The average value of the overall TLX was 4.48: which means, on average, we were able to get the patients in the best conditions for training their cognitive ability. As expected, the proposed exercise did not demand any physical effort from the participants. Also, frustration only accounted for 2.61 over 10. This result was possible not only because the exercise was tailored to the patient (effort was equal to 5.4) but also because of the therapist’s ability to offer them the assistance that best suited their needs. Finally, as a complementary result, performance was 6.78: which means the patients estimated that they had performed well, but not perfectly. A higher value would have told us that the exercise and/or the assistance offered were not appropriate. These two values confirm that, on average, the aim of the study was achieved.

Concerning the objective measures, we evaluated the average standard deviation of participants’ performance over the six sessions. In other words, we aimed at computing the ability of the therapist to keep the patients’ performance constant during the six sessions. The average number of attempts for the patients was 11.55, and the overall standard deviation was 2.34.

Despite the fact that we did not run any statistical significance analysis, we can observe from the results of the NASA test and the average standard deviation of the patients’ performance that, on average, the therapist managed to provide tailored assistance to keep the patients’ performance constant with a dispersion around the mean of 2.34. Finally, it emerged that the patients did not manifest any feeling of discomfort.

## Study with the robot therapist to evaluate CARESSER

With this study, we aimed at evaluating whether CARESSER was capable of learning a patient-specific assistive behaviour by keeping their performance constant during the six sessions. The robot’s assistive behaviour was defined in the observational study (see Sect. 4.1), and the different levels of assistance that CARESSER was asked to learn are shown in Table 1 (Column Learnt).

Regarding the experimental design, as stated in Sect. 6, the same participants that participated in the study with the human therapist (see Sect. 7) were also participating in this study.

It is important to note that the timing of the assistance was not directly learnt by CARESSER, but we estimated it from the previous study, averaging the time the therapist waited before offering assistance during the sessions. Finally, as reported in Table 1, for the appraisal support, we had a predefined set of sentences from which we selected one, depending on the number of attempts made by the patients.

### Study introduction

We present a study in which a TIAGo robot was asked to administer a cognitive exercise to patients suffering from mild dementia and mild cognitive impairment, offering them assistance and hints (see Table 1).

For all the patients (except one), this was the first time they had interacted with a robot, and hence, we offered them a few minutes to ask questions about the robot and their first impression. As in the previous study, the participants played with the robot for six sessions.

In the first session, the robot gave assistance to the patients by using the policy it had learnt with the data gathered from the previous study (see Alg. 3, *s* = 0). During each session, the therapist provided his feedback on the robot’s assistive behaviours by evaluating the correctness of the level of assistance offered through a report sheet. At the end of each session, while the therapist prepared the tokens for the next round, the experimenter ran CARESSER, which estimated the new policy for the next session (see Alg. 3). CARESSER, depending on the current performance of the patients with respect to their average performance from the previous study, could opt to challenge them more (if they performed better than expected) or, on the contrary, to support them more (if they performed worse than expected). The two different strategies have been extensively described in Sect. 5.2. This stage is where the personalisation occurred and in which a policy-specific to the patient was actively learnt by the robot, session after session.

### Example of a session

The videos^4^ show an example of a session between the TIAGo robot and a patient. As can be observed, the robot intervenes to provide assistance or guidance to the patient after a token is placed (correctly or incorrectly) or a timeout occurs.

In the first video, the patient plays with the robot for the first time (*S* = 1). It can be observed, as in the middle of the exercise (when the patient has to place the third token), the patient strives to find the correct one: therefore, the robot gradually increases its assistance level, eventually placing the correct one on the patient’s behalf.

In the second video (*S* = 3), the patient leverages SOCIABLE to know whether the grasped token is incorrect and the interactions are faster. Note that even though the patient plays with more confidence in this session, she struggles to find the last token, and eventually the robot moves the token on her behalf.

In both the videos, it can be noticed how the patients are also asked to remember the location of the tokens, as if they move it in an incorrect location, they need to place it back in its original location.

### Results and discussion

In this section, we present and discuss the results of this study.

Firstly, we assessed whether H1 stands or fails by evaluating the therapist’s feedback on the robot’s assistive behaviour (see Sect. 8.3.1). H1 addressed RQ1a.

Secondly, we evaluated H3 and H4 by comparing the patients’ performance when they interacted with the human therapist, the robot therapist, or when simulated by using GOAL (see Sect. 8.3.2). H3 and H4 helped in assessing RQ1b.

Thirdly, we evaluated the cognitive workload of patients when playing with the human therapist and the robot therapist (H2, see Sect. 8.3.3). Finally, we measured whether CARESSER was able to keep the patients’ performances constant during the sessions better than the therapist (H5, see Sect. 8.3.4). H2 and H5 provided arguments to tackle RQ1c.

#### Therapist evaluation

Aiming to evaluate the therapist’s feedback on each robot’s assistive action, a report sheet was provided to the therapist at the beginning of each session. Then, he was requested to fill it in with a score between 1 and 5, as explained in Sect. 6.8. Furthermore, he was also asked to report any kind of behaviour or action that the robot performed incorrectly in terms of interaction modalities and timing or that it did not execute. Therefore, for each patient, the therapist filled in six evaluative reports corresponding to each session the patient played with the robot.

**Fig. 8:**
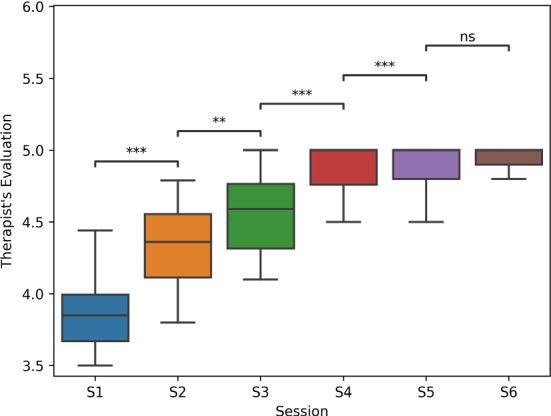
The figure shows the average score assigned by the therapist to each session (n.s. denotes *p* > .05, * denotes .01 < *p* < .05, ** denotes .001 < *p* < .01, and *** denotes .0001 < *p* < .001)

Figure 8 shows the average evaluation of the therapist during the six sessions for the 22 patients. As can be observed, the rating assigned by the therapist to the robot’s behaviour soared session after session, eventually reaching the top score already at the third session for some of the patients. However, it seems that already starting from the fourth session, the robot’s behaviour is almost as expected by the therapist.

To test our initial hypothesis H1, we ran Friedman’s test to compare the effect of a session (independent variables), on the therapist’s evaluation of the robot’s behaviour (dependent variable) during the six sessions played with the robot. There was a significant effect of session on therapist’s evaluation score, $\chi ^2(5)=68.44$, $p<0.001$. Because the significance level is below 0.05, we can study the individual p-values to find out which of the individual variables are statistically significant.

Post hoc multiple comparisons using Conover adjusted by the Hold method showed: a significant difference between the rating provided by the therapist after S1 (*M* = 3.51, SD = 0.52) and S2 (*M* = 4.13, SD = 0.45) with $p<0.001$, a significant difference between the rating provided by the therapist after S2 (*M* = 4.13, SD = 0.45) and S3 (*M* = 4.38, SD = 0.31) with $p<0.01$, a significant difference between the rating provided by the therapist after S3 (*M* = 4.38, SD = 0.31) and S4 (*M* = 4.46, SD = 0.47) with $p<0.001$, a significant difference between the rating provided by the therapist after S4 (*M* = 4.46, SD = 0.47) and S5 (*M* = 4.78, SD = 0.26) with $p<0.001$, and finally no significance difference between the rating provided by the therapist after S5 (*M* = 4.78, SD = 0.26) and S6 (4.77, SD = 0.19) with $p=0.15$.

Overall, there is a clear trend that shows that the therapist’s evaluation rating, on average, increased during the sessions. If we have a close look at the box plots, it is clear that in the first sessions (S1, S2 and S3), the robot was still learning the correct policy for the given patient; that is, the therapist’s rating was still far from his desired behaviour. The closer we get to the sixth session, the better the robot’s behaviour was, eventually getting a score that did not differ from the maximum score of 5. From these results, it seems that the robot only needed the first four sessions to fulfil the therapist’s preferences and therefore to converge to the expected robot’s behaviour at the fifth session, which highlights the fact that the therapist did not find any difference in the robot’s social behaviour in comparison with the last session. We can then conclude that our initial hypothesis H1 is supported by our findings.

#### Patients’ performance comparison: human therapist versus GOAL versus robot therapist

In this section, we aim at comparing the average participants’ performance during the six sessions whether the assistance was offered by the human therapist, the agent therapist generated from GOAL (shortly the simulator), or the robot therapist.

The main motivation for including GOAL was to evaluate whether and to what extent the simulator which employs a hybrid approach (data and knowledge-driven) was capable of estimating the patient’s evolution over the sessions. As GOAL estimates the patients’ performance over hundreds of runs, we compared this value with the average patients’ performance in the two studies.

**Fig. 9:**
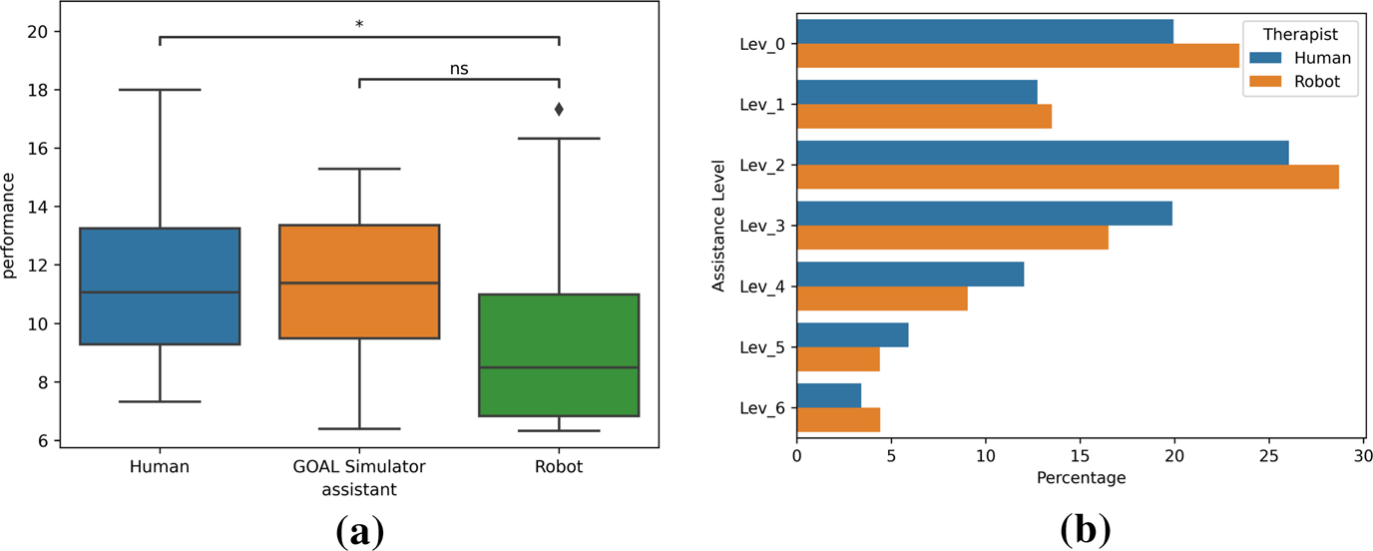
**a** How the performance of the patients (*y*-axis) changed according to who provided them with assistance (*x*-axis). It is important to note, that in simulation, both the therapist and the patients were simulated according to the generative models described in Sect. 5.1 (n.s. denotes *p* > .05, and * denotes .01 < *p* < .05). **b** The average assistance for each level of Table 1 offered by the human therapist (top blue bar) and the robot therapist (bottom red bar), respectively. (Color figure online)

The comparison is shown in Fig. 9a. To test our initial hypotheses H3, we ran a multi-linear regression model with those who provided assistance (human or robot therapist) as independent variable, including the patients’ therapy time as an additional predictor, which is defined as *morning* or *afternoon*, with the first meaning the patient did the experiment with the therapist, either human or robot, in the morning and the latter meaning the patient did the experiment with the therapist, either human or robot, in the afternoon. The overall model fit was $R^2=0.41$ ($F(3,40)=9.28$, $p<0.0001$). As the overall F-test value is less than the significance level of 0.05, we can study the individual p-values to find out which of the individual variables are statistically significant.

Results showed that there was a significant effect of the variable therapist on the users’ performance; that is, when the therapist is a human, patients had overall worse performance ($\beta =1.06$, $p<0.001$). Furthermore, patients that had the cognitive session with the therapist in the morning (first three patients) had better performance ($\beta =-0.60$, $p<0.05$) compared to those who had their session late in the day, regardless of who provided them with assistance. Finally, patients that interacted with the human therapist in the morning (first three patients) had better performance ($\beta =-0.48$, $p<0.05$) compared to the patients that interacted with him in the afternoon (last three patients). These results seem to confirm H3 whereby the human therapist might have reduced his effectiveness of providing tailored assistance over time presumably as a result of boredom or tiredness.

To gain more insight into what supports hypothesis H3, we decided to plot also the average percentage of each level of assistance, offered by the human therapist and the robot therapist in the two studies to assess whether the performance of the patients was caused by the higher level of assistance offered by the robot therapist. As illustrated in Fig. 9b, overall, the robot provided less informative assistance than the human therapist. Specifically, Lev_3, Lev_4, Lev_5, and Lev_6 were offered by the robot therapist 16.5%, 9.04%, 4.4%, and 4.42% of the time, respectively. Differently, the human therapist provided those levels 19.88%, 12.03%, 5.92%, and 3.41% of the time, respectively. On the other hand, the robot therapist provided Lev_0, Lev_1, and Lev_2, 23.42%, 13.5%, and 28.7% of the time compared to the human therapist (Lev_0=19.88% Lev_1=12.47% and Lev_2=26.05%). This result provided an additional argument to support our findings with respect to hypothesis H3. Not only did we demonstrate that the patients interacting with the robot therapist obtained better results, but also that they needed less assistance. We argue that this was possibly due to the presented framework, CARESSER, which is accountable for generating the correct levels of assistance according to the patient’s cognitive model that is actively updated during the six sessions.

To test our initial hypothesis H4, we ran a simple linear regression with those who offered assistance (simulator, robot therapist) as predictor (independent variable), controlling for patient’s performance (dependent variable). Note that we used a regression coding in which the robot therapist is considered as the reference group, whose value corresponds to the average patients’ performance when interacting with the robot therapist (*M* = 9.42, SD = 2.82).

Specifically, the results shown in Fig 9a revealed that the difference between patients’ performance over the six sessions when interacting with the robot therapist (*M* = 9.42, SD = 2.82) and when the patients’ interactions with the therapist were simulated (*M* = 10.68, SD = 2.75) is not significant, indicating that patients, on average, did not perform differently whether they were simulated or interacting with the robot ($R^2=0.04$, $F(1, 43)=1.907$, $\beta =1.25$, $p=0.17$). Therefore, the findings support H4. As expected, CARESSER, given its hybrid method, which combines data- and knowledge-driven approaches with active learning is capable of estimating the patients’ performance with no significant difference from the real one that took place when they interacted with the robot.

**Fig. 10:**
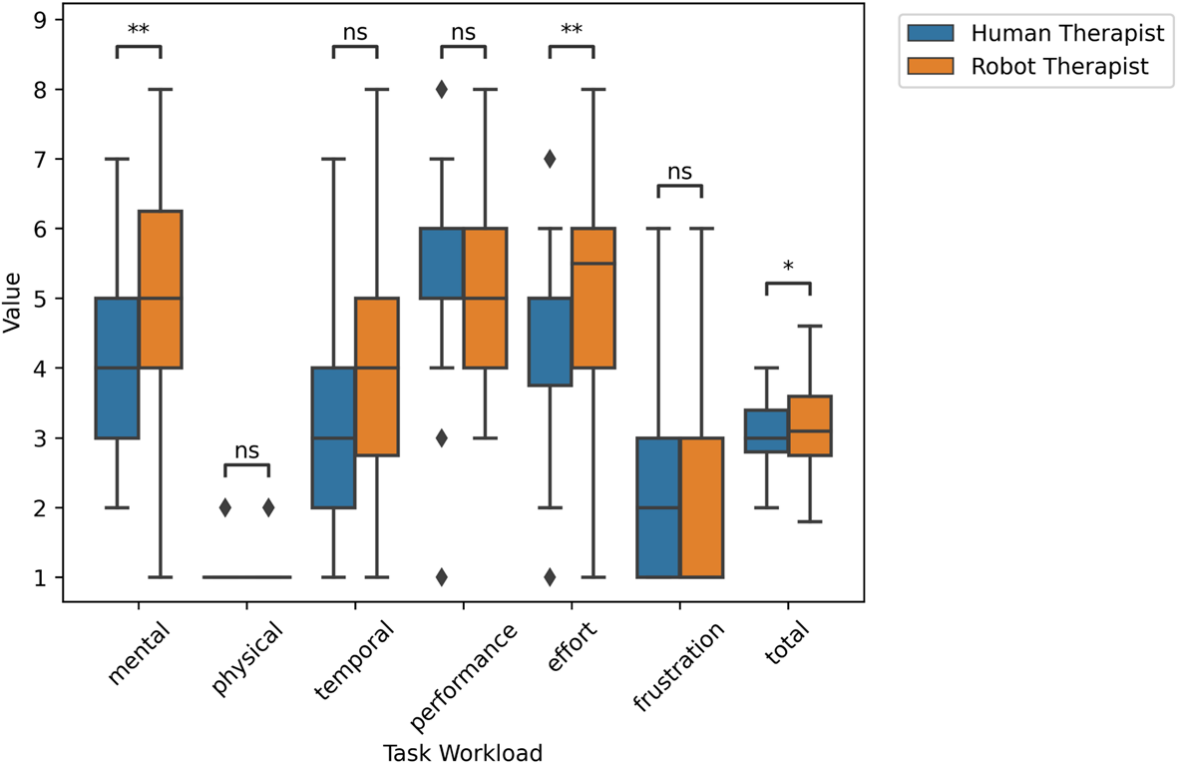
The figure shows the results of the NASA TLX test when the patients interacted with the human therapist and the robot therapist, respectively (n.s. denotes *p* > .05, * denotes .01 < *p* < .05, and ** denotes .001 < *p* < .01)

**Table 6:** Results of the NASA TLX test from the robot therapist study

	Mental	Physical	Temporal	Effort	Performance	Frustration	Overall
*M*	6.27	1.45	4.5	6.54	6.4	2.32	4.83
SD	2.19	0.46	2.03	1.7	2.07	1.57	1.05

#### Task workload comparison: human therapist study versus robot therapist study

In this section, we aim at evaluating whether and to what extent patients had perceived any difference in terms of cognitive workload in the two studies by comparing the results of the user study with the human therapist (see Table 5) and that of the robot therapist (see Table 6). The comparison is shown in Fig. 10. To test our initial hypothesis H2, we ran a simple linear regression with each dimension of the TLX as predictor (independent variable), controlling for patients’ perception of the task workload (dependent variables). With the regression coding being used, the human therapist was coded as the reference group (the intercept). Specifically, the results shown in Fig. 10 revealed that the mental demand, that is, how much thinking and deciding was required to perform the task, in the two studies was significant, indicating that the patient struggled a bit more when interacting with the robot therapist ($\beta =1.11$, $F(1,110)=8.49$, $pi<0.05$). In the case of physical demand, we did not find any statistical significance ($\beta =-0.04$, $F(1,110)=0.23$, $p=0.62$). The same findings were seen for temporal demand ($\beta =0.24$, $F(1,110)=0.44$, $p=0.5$), performance ($\beta =-0.24$, $F(1,110)=0.6$, $p=0.41$), and frustration ($\beta =-0.29$, $F(1,110)=0.99$, $p=0.32$). In the effort dimension, the NASA TLX value showed a significant difference, with the mean value of when interacting with the human therapist being smaller than when they interacted with the robot therapist ($\beta =1.004$, $F(1,110)=8.07$, $p<0.001$). Finally, the overall cognitive workload showed a significant difference in rating, indicating that when patients interacted with a robot therapist, they received a higher rating than when they interacted with a human therapist ($\beta =0.35$, $F(1,110)=4.56$, $p\le 0.05$).

We argue that the differences that emerged along these dimensions occurred because, in the robot therapist study, the robot’s behaviour changed according to the patients’ performance. That is to say, the robot therapist on average provided less informative assistance than the human therapist, as shown in Fig. 9b. Additionally, in the robot therapist, patients were more focused and engaged in the task as reported in the therapist’s reports. That can be appreciated by their performances, which were on average better than when interacting with the human therapist (see Fig. 9a). This additional effort requested by the robot was perceived and rated. Despite the higher scores on these dimensions, the differences are still not so relevant as to deduce any negative effect on the patients’ cognitive workload. Instead, these results offer additional evidence of the adaptivity of the robot and of its capability to challenge the participants when needed.

We can conclude that the findings did not fully support our experimental hypothesis H2, and therefore, the task workload of patients interacting with the therapist was lower than when they interacted with the robot for some of the TLX’s dimensions. However, results do not provide any evidence that the robot’s assistance had a negative impact on the participants; on the contrary, it seems to have prompted them to perform better.

#### Challenge point comparison: human therapist versus robot therapist

In order to assess whether the robot was capable of keeping the patients’ performance constant (according to challenge point theory (Guadagnoli and Lee 2004)), we computed, for each patient, the standard deviation of their performance over the six sessions. Additionally, we compared it to the standard deviation of the same patients obtained in the previous user study when they interacted with the human therapist.

To test our experimental hypothesis H5, we ran a simple regression in which the therapist (who offered assistance to the patient) is the predictor (independent variable), controlling for patient’s average standard deviation of their performance (dependent variable). With the adopted regression coding, the human therapist is considered as the reference group, whose value corresponds to the patients’ average standard deviation of their performance when they interacted with him.

**Fig. 11:**
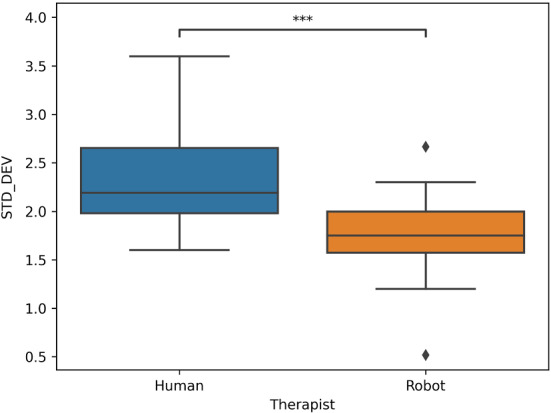
Results of the human therapist and the robot therapist to keep the patients’ performance constant over the six sessions. The standard deviation between the six sessions is employed as an evaluation metric (*** denotes .001 < *p* < .0001)

The results are shown in Fig. 11. As indicated by the box plot, the *SD* value was lower when patients interacted with the robot (*M* = 1.73, SD = 0.45) compared to the therapist (*M* = 2.34, SD = 0.54). Specifically, the result revealed that the ability of the robot therapist to keep the patients’ performance constant was significant with respect to the human therapist, indicating that the robot offered assistance in a more appropriate way ($R^2=0.27$, $\beta =0.6$, $F(1,44)=16.86$, $p<0.001$). Alternatively stated, the robot accomplished the objective to keep the patients’ performance constant better than the human therapist did. Therefore, our findings supports H5.

In order to evaluate further how CARESSER accomplished the task of tailoring its assistance over the sessions, inspired by the work of Park et al. (2019), we computed the correlation between two policies in terms of state-action. In other words, we aim to assess to what extent the robot’s policy for one patient is unique with respect to all the others. Therefore, we compute the action-match value as follows:

2 $$\begin{aligned} a_\mathrm{{match}}=\frac{1}{N* \left| S \right| }\sum _{r=1}^{N}\sum _{s=0}^{S}\left[ \max _{a}Q_{x}^{r}[s,a] = \max _{a}Q_{y}^{r}[s,a] \right] \end{aligned}$$

where *N* is the number of sessions and |*S*| is the number of states and $Q_{x}$ and $Q_{y}$ are two policies.

**Fig. 12:**
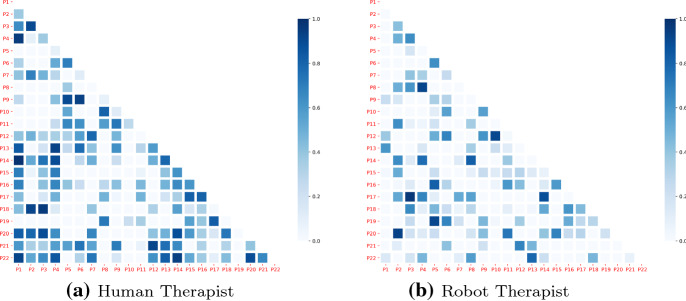
The two correlation matrices illustrate the rate of the number of matching actions between two policies, summed up after each of the six sessions for each patient when the assistance is provided by the human therapist (**a**) and the robot therapist (**b**). Low correlation indicates more personalisation

We report the results for the human therapist and the robot therapist in Fig. 12. As shown by the correlation matrix depicted in Fig. 12a, overall, the human therapist’s policies did have quite a lot in common, meaning the therapist struggled to adapt the assistance to a given patient and he might apply the same assistance pattern for different patients. On the contrary, it can be noticed from Fig. 12b that the robot therapist did tailor its assistance to each patient. The number of cells in which there is a high correlation with other policies is much smaller compared to that in which the human therapist assisted the patient. This behaviour was achieved as a result of the active learning stage included in the CARESSER framework. As a matter of fact, in each session, the framework evaluated, based on the patients’ performance history, whether it needed to challenge or offer them more help. This continuous adaptation to the patients’ performance enabled the robot to offer them tailored assistance.

## General discussion

This study demonstrated the feasibility of learning a personalised social robot policy for 22 patients affected by mild dementia and cognitive impairment, who played a cognitive exercise with the assistance of a robot in a fully autonomous fashion. This was achieved by adopting a hybrid approach that combines data gathered from the therapist’s demonstrations and his expertise to build patients’ and therapist models and actively reshape them during the sessions. We demonstrated that CARESSER converged to the therapist’s expected behaviour after the fourth session (supporting H1). We also provided evidence that CARESSER succeeded in maintaining the patients challenged according to the challenge point theory, that is, keeping their performance constant during the six sessions by actively adapting to the patients’ skills (supporting H5). A key component of CARESSER is GOAL, a simulator which was accountable to generate episodes according to the cognitive model of the patient and the therapist. It actively reshapes the robot’s behaviour on the basis of the patients’ performance. We argue that this is a piece of very valuable evidence that proves the effectiveness of the CARESSER framework, which did not only reproduce the human therapist’s policy according to the gathered data and the therapist’s initial setting but also actively tailored its assistance based on the specific individual needs during the sessions.

Another interesting outcome was that the patients’ performance in the human therapist study and the robot therapist study was significantly different (supporting H3), albeit we did not find any difference between the simulated patients generated with GOAL (supporting H4). Indeed, patients committed fewer mistakes and received less supportive assistance when interacting with the robot therapist, that employed CARESSER, than when they interacted with the human therapist. Furthermore, the patients who did the experiment with the robot in the afternoon had worse performance than those who did it in the morning. On the other hand, the perceived cognitive demand of patients when interacting with the robot was higher than when they interacted with a human therapist with respect to three of the six dimensions of the TLX test: effort, mental demand, and overall cognitive workload (partially supporting H2). These results seem to confirm that (i) the therapist might have experienced some form of boredom or tiredness during the daily sessions with the patients, (ii) patients were more committed when the robot administered the exercise, and also because of the novelty effect that might have impacted their engagement (Gross et al. 2011). These findings are also in line with Pino et al. (2020), who showed that older adults affected by mild cognitive impairment that received cognitive training to enhance memory and attention through the humanoid social robot (NAO) achieved more visual gaze, less depression, a lower level of anxiety, and a higher level of perceived memory efficiency compared to the same interactions with the therapist.

Nonetheless, these insights can only be considered as the outcome of a preliminary analysis conducted with only one therapist, and yet they need to be validated with a larger sample and with more therapists.

### Lessons learnt

This section highlights some of the more critical lessons learnt throughout the development, deploying and evaluation of a fully autonomous social robot aimed at providing cognitive training to patients with cognitive deficits. In addition, some valuable lessons were gleaned from interviews with the healthcare professionals involved in the studies. We divide them into robot’s functionalities and experimental design.

Concerning the robot’s functionalities, we found that:

- Patients were keen to interact verbally with the robot, and, despite having been told that it was not able to understand, most of them strove to talk to it. Therefore, researchers should find a way to enable this functionality in the robot.
- The robot’s action “ask caregiver intervention”, which aims at restoring the situation to a state that the robot is capable of managing again, was very helpful. Patients sometimes got lost and made unexpected actions. This capability helped to avoid the system getting stuck and the exercise being reinitialised. On the other hand, patients did not feel responsible or ashamed of having broken the system because of their memory impairment.
- Patients were very responsive and quite amused by the robot’s capability to blink its eyes and show some facial expressions. The healthcare professionals noted that this feature was key in keeping them concentrated and engaged during the entire duration of the study. Therefore, we recommend researchers to bear in mind the opportunity to deploy this feature into a robotic system.
- The politeness of the sentences and the pro-social behaviour of the robot were very appreciated (see Sect. 4.4). Some patients explicitly pointed out how much they liked the way the robot talked to them.
- Gesture as an interaction modality was very effective. Older adults in general struggle to follow speech and have more trouble comprehending the person they are listening to. Hence, the ability of the robot to provide assistance through its arm was very well accepted and deemed crucial by the healthcare professionals. Indeed, the combination of the three modalities (voice, gesture, and facial expression) strengthened the message and provided the patients with visual and audio feedback.
- Between the social assistive levels offered by the robot, reminding of the rules was the most effective when the patient got stuck. This assistance would not have been possible if we had not carried out the observational study and not involved the stakeholders in the design of the robot’s behaviour.
- Patients did not show any specific emotion during the observational study and the human therapist study. Therefore, it was not worthwhile to include patients’ emotions or even facial expressions as an additional driver to our framework in the robot therapist study. What we and the healthcare professionals realised is that they did show some emotions related to their feelings during the exercise, but the emotions were more related to their body movements and utterances.

Regarding the experimental design, we found that:

- The warm-up time, dedicated to the patients getting to know the robot, was crucial for the perfect outcome of the experiment. Healthcare professionals realised how valuable it was to leave them interacting and asking questions about the robot. This is especially true for older adults who are not used to technology and have never seen a robot before. In the human therapist study, when they were asked how they thought the robot would look, most of them could not even guess, as they did not have the ability to envisage such a thing.
- Asking the patients to move back the token to the same location when it was incorrect, was complicated for most of them, as they forgot where it was located. We realised that, after a few attempts, the robot should assist the patients more and place the token back on their behalf.
- Apart from the degree of impairment, background and educational level counted very much in selecting the correct exercise. Patients with lower MMSE than others were capable of solving a more complex exercise because of their background. Thus, allowing the therapists to customise the exercise is key for providing effective cognitive therapies as well as to keeping the patient engaged during the exercise.

### Limitations and outlook

Despite our results demonstrating the effectiveness of CARESSER in the proposed scenario and of deploying that learning system in a social robot in a fully autonomous fashion, some limitations should be noted and motivate future work. We classify them into methodological and developmental limitations.

Regarding the methodological limitations, we note the following:

- The number of participants involved in the experiment is in line with most real-world studies, with the exception that here participants were patients with cognitive impairment whose recruitment is usually very complicated and even more during the pandemic for COVID-19. Despite the very promising results, the insights collected in this study need to be validated on a larger population.
- On the same line, we believe that this study might suffer from what is known as the novelty effect, so a longer study should explore the patients’ engagement as well as their acceptance of the robot over time.
- This study relied on a single therapist, as in the work of Senft et al. (2019). Hence, the obtained results are bounded to the therapist involved in the study. However, we argue that a therapist’s approach is quite unique in terms of interaction styles, assistance, and communication with the patient, and therefore, we speculate that it is reasonable to assess it firstly with one therapist and then try to generalise it to more than one. Future work should concentrate on replicating the study with different therapists in order to validate whether the same conclusions can be drawn. We expect to find differences in their approaches but overall not significant in terms of outcome. Nonetheless, having more than one therapist would require to redesign our framework to reason about the demonstrations provided by different therapists.
- The order in which the user studies were conducted could not be changed or randomised; therefore, there might be a learning effect. Nonetheless, as already highlighted in Sect. 6, we argue that it is very unlikely to occur. Apart from the incapability of the patients to remember those exercises after one week, the cognitive exercises we proposed are inspired to those they are used to playing with the therapists during their daily therapy, and hence, it was nothing new to them.

Concerning the developmental limitations, we mention the following:

- The perception system was only based on contextual information, without including any other information coming from the patient. Understanding how patients feel and perceive the exercise is very important. Regarding patients’ facial expressions, we did not notice any change in terms of valence and arousal by analysing the videos. Additionally, most of the current software for emotion recognition does not work very well with older adults, mainly because of the bias in the dataset (older adults are underrepresented) and to age-related structural changes in their faces (Caroppo et al. 2018). However, we did observe that patients used their body very often to communicate their feelings. Therefore, an upper-body tracker combined with speech recognition might provide additional information to our framework and enhance its ability to furnish the correct assistance. Another option would be to adopt sensors such as galvanic skin response (GSR), heart rate (HR), or portable electroencephalography (EEG) that, in different ways, provide bio-metric measures of an individual.
- In the way the exercise was designed, it was complicated for the robot to intervene while the patient was playing, because of safety reasons, every time the patient picked a token, the robot stopped its action. As the exercise attempted to train memory and attention, patients were very focused on the board, sometimes missing eye-contact with the therapist (human and robotic). We believe that a more collaborative exercise in which the robot acts as a peer might engage the patients more and enable the burden of the completion of the exercise to be shared between the patient and the robot.
- We intentionally did not include speech recognition, despite the willingness of the patients to interact by voice with the robot. This is because technology is not ready yet, especially in recognising older adults’ voices (Pou-Prom et al. 2020; Law et al. 2019) and a failure in the recognition might generate frustration and unpleasant feelings. However, being able to integrate speech recognition and dialogue management would ensure a more natural interaction.
- The speech utterance rate of the robot could not be modified. The healthcare professionals noticed that in most of the cases the pace at which the robot spoke was too fast for the patients. Furthermore, the robotic voice tended to limit the patients’ understanding. This functionality should be integrated into the robotic platform itself, as it would offer a higher degree of customisation of technology based on the user’s individual needs.
- The robot mastered very well which assistance to offer and to whom, but the CARESSER framework did not learn when to offer it. As stated, we only set it by averaging the human therapist’s intervention time. Learning when to offer assistance is a key aspect on which we will focus our future work.
- We validated CARESSER’s effectiveness in a low-dimensional task. In the future, we aim to assess whether the current framework could be employed in more complex tasks. In the case of a higher-dimensional task, we could define patients’ profiles or Personas based on the therapist’s expertise. In doing so, we could already have part of the questionnaire filled based on this a priori information. This step might help to speed up the initialisation, reducing the burden of filling in the questionnaire from scratch for the therapists.
- The cognitive models of the patient as well as of the therapist (human or robotic) have been designed using two different BNs. It might be worth exploring whether a dynamic BN would be a more elegant solution for modelling the same problem in only one network.
- The logic used to decide when to change the simulator strategy (“challenging” or “helping”) based on the performance of the patient was limited and there might be some situations in which the simulator fails. One possible situation is when the patient performs much better than expected (they complete the exercise with no mistake). Thus, we run GOAL aiming at challenging it more, but the patient’s performance is still outstanding. Eventually, we can end up in a situation in which the robot therapist does not provide any help (lev 0) and the patient still performs much better than expected. The same can happen when the patient performs much worse than expected. In the proposed scenario, the exercise’s complexity is fixed, hence the only manner of intervening to affect the patient’s performance is by changing the levels of assistance provided by the therapist. It would be of interest to implement an intervention of the therapist also on the complexity of the exercise that might prevent the simulator from failing in these situations.
- The MCE algorithm requires complete knowledge of the environment, which might be unfeasible in some real-world scenarios. However, model-free versions of MCE already exist and might be a valid replacement (e.g. Martinez-Gil et al. 2020).

## Conclusion

This paper demonstrates the potential of the CARESSER framework to enable robots to actively learn from experts’ demonstrations (data-driven approach) and expertise (knowledge-driven approach). In this way, we can guarantee short-term adaptivity and personalisation. Adaptivity as the system constantly shapes its behaviour according to the current state and its previous history. Personalisation, as the system tailors its behaviour according to the patient it interacts with. These two capabilities are especially useful in socially assistive robotics, in which robots are requested to interact with vulnerable populations and thus must be able to meet the patient’s individual needs. An important aspect of the proposed framework is that it learns directly from therapists, bypassing two critical stages from standard approaches: (i) the design of a reward function that enables personalisation (forward reinforcement learning) and (ii) the continuous presence of a human expert that supervises the robot’s actions (interactive reinforcement learning). The first stage is overcome by using an IRL method that learns a patient-specific reward function, instead of having to code it manually for each patient. The second stage is addressed by using CARESSER, which through the GOAL simulator generates episodes according to the actual patient’s performance, by evaluating whether to adjust the current policy (challenge or help) when the patient did not perform as expected or keep it as it is.

The implications of this study are threefold: firstly, we provide evidence that CARESSER can be applied successfully in a real use-case assistive scenario, whose participants are patients affected by dementia and cognitive impairment. The framework is not only capable of generating a policy that takes into account the therapist’s behaviour and a priori information of the patients’ cognitive abilities, but also of actively reshaping its policy during the sessions, eventually matching the therapist’s preferences (RQ1a). Secondly, we prove how the GOAL simulator, as part of the CARESSER framework, correctly approximates patients’ performance and contributes to the generation of an appropriate policy for a given patient (RQ1b). Finally, we showed how CARESSER was competent in keeping the patients challenged, that is, keeping their performance constant over the sessions, by maintaining an updated cognitive model of the patient it was interacting with and offering tailored assistance based on the patients’ needs (RQ1c). These three implications can pave the way to new AI approaches, that learn by leveraging human experts’ demonstrations as well as their expertise, thus speeding up the learning process of the algorithm and avoiding undesired states. Furthermore, as in the work of Winkle et al. (2020), we highlight the importance of including the stakeholders in the design process as well as in the automatisation of the robot assistive behaviour.

We believe these results contribute to addressing some of the challenges of socially assistive robotics: (i) designing and deploying a robot with appropriate social behaviour in a fully autonomous fashion in a real-world scenario; (ii) coping with the complex multi-modality nature of the interactions; and (iii) learning effective social assistive behaviour from a limited number of interactions.

In summary, this work shows possible directions for future development of autonomous personalised social robot behaviour in an assistive context.

## Preliminaries

In this section, we briefly introduce the Markov decision process (MDP) and the fundamentals of RL and IRL, with a focus on the foundations of the causal maximum entropy (CME) method that will be used in this work.

### MDP

An MDP is a tuple $\left\langle S, A, P, R, \gamma \right\rangle$. *S* is a finite set of states, *A* is a finite set of actions, *P* is a state-transition probability matrix defined, such as, $P_{ss'}^{a}=\mathbb {P}\left[ S_{t+1}=s' | S_{t}=s, A_{t}=a \right]$. In other words, it defines the probability that in state *s* if the agent performs the action *a*, it will bring the agent to state $s'$. *R* is a reward function defined, such as, $R_{s}^{a}= \mathbb {E} \left[ R_{t+1} | S_{t}=s, A_{t}=a \right]$. In other words, it defines the expected immediate reward received by the environment at the transition from state *s* to state $s'$ by performing action *a* and transition probability *P*. Finally, $\gamma$ is the discount factor.

At each time step *t* (if time is discrete), an agent chooses an action $a_{t} \in A$ which in turn causes a transition from state $s_{t}$ to some successor state $s_{t+1}$ with probability *P*. The agent receives a scalar reward (positive or negative) $r_{t+1}$ for choosing action $a_{t}$ in state $s_{t}$. One important property of the MDPs is that the future is independent from the past given the present. More formally, a state $S_{t}$ is Markov if and only if $\mathbb {P}\left[ S_{t+1}|S_{t} \right] = \mathbb {P}\left[ S_{t+1}|S_{1}, ...,S_{t} \right]$.

The action $a_{t}$ performed by the agent at state $s_{t}$ is defined according to a policy $\pi _{t}$, which is a mapping from states to probabilities of selecting each possible action (stochastic policy).

### RL

Reinforcement learning methods define how an agent changes its policy according to its experience. The agent’s objective is to find a policy that maximises the accumulated sum of discounted future rewards over time, such as $R_{t} = \sum _{t=0}^{\infty } \gamma _{^{t}}r_{t+1}$. $\gamma \in [0,1]$ determines how much importance we assign for future reward. If $\gamma$ is closer to zero then the agent is blind, in the sense that, it will only consider immediate rewards. On the other hand, if $\gamma$ is closer to 1, then the agent is more farsighted.

Almost all the RL algorithms involve estimating value functions that assess “how good” it is for the agent to be in a given state. More formally, we can define a value function $v_{\pi (s)}$ as:

3 $$\begin{aligned} v_{\pi }(s)= \mathbb {E}_{\pi }\left[ G_{t}|S_{t}=s\right] =\mathbb {E}_{\pi } \left[ \sum _{k=0}^{\infty }\gamma ^{_{^k}} R_{t+k+1}\mid S_{t}=s \right] \end{aligned}$$

$V_{^\pi }$(s) denotes the expected value of a random variable in state *s* given that the agent follows policy $\pi$.

In the same way, it is possible to define the value of taking action *a* in state *s* under a policy $\pi$, denoted $q_{\pi }(s,a)$, as the expected return starting from *s*, taking the action *a*, and thereafter following policy $\pi$.

4 $$\begin{aligned} q_{\pi }(s,a)= \mathbb {E}_{\pi } \left[ G_{t}|S_{t}=s, A_{t}=a \right] = \mathbb {E}_{\pi } \left[ \sum _{k=0}^{\infty } \gamma ^{_{^k}} R_{t+k+1} \mid S_{t}=s, A_{t}=a \right] \end{aligned}$$

Solving a reinforcement learning problem means, roughly, finding a policy that maximises the expected sum of discounted future rewards over the long term. In other words, we are interested in finding a policy $\pi ^{*}$=${\text {*}}{arg \max }_{a}q_{\pi }(s,a)$.

According to Heidrich-Meisner et al. (2007), RL can be classified into critic-only, actor-only, or actor-critic methods. The main idea behind critic-only methods is that the agent tries to learn an optimal value function and then it derives an optimal policy. On the contrary, actor-only methods search for the best action directly in the policy space. The third method is obtained combining both the previous approaches. Essentially, the critic monitors the agent performance and determines when the policy should be changed and therefore improved.

### IRL

Inverse reinforcement learning (IRL) assumes that the MDP is given as a tuple $\left\langle S, A, P, \gamma \right\rangle$ without the reward function R. In IRL, we are provided with a set of observed behaviours (known as trajectories) $D={d_{0}, d_{1}, ..., d_{n}}$, where $d=((s_{1}, a_{1}), (s_{2}, a_{2}), ..., (s_{m_{h}}, a_{m_{h}}))$ is a state-action sequence of length h which is assumed to be samples of policy $\pi _{D}$. In IRL, the objective is to learn the unknown function R that caused an agent to produce those behaviours. In contrast to RL, which aims at learning a policy from samples of a reward function, the IRL problem attempts to learn a reward function from samples from a policy.

Within IRL, three main methods can be identified. Maximum-margin methods that tackle the problem of finding a reward function that is as good as possible compared to that of the expert’s policy by a margin. Feature expectation matching methods that attempt to find a policy that generates features similar to those generated by the expert’s policy according to the maximum entropy principle. Finally, Bayesian approaches, which encode the reward function as the prior which is then combined with a likelihood function for expert demonstrations (the evidence) to form a posterior over reward functions which is then sampled using Markov Chain Monte Carlo (MCMC) techniques.

In this article, we formulate IRL as a maximum causal entropy (MCE) task (Ziebart et al. 2010). MCE as other methods solve IRL by way of feature matching. This methods consider the existence of a vector $\phi$ of feature functions $\phi _{i}: \mathbf {S \times A} \mapsto {\mathbf {R}}$. It is assumed that the feature functions $\phi$ measure properties that are relevant for the process of deriving a reward function.

If we assume that the reward function is obtained by linear combination of the feature functions, the feature expectation vector of a policy $\pi$ fully describes the reward function obtained by acting using this policy. In feature matching methods, the goal of the IRL is to optimise the reward function to generate a policy $\pi$ with a feature expectation vector ${\mathbf {f}}_{\pi }$ that satisfies ${\mathbf {f}}_{\pi } = {\mathbf {f}}_{D}$, where ${\mathbf {f}}_{D}$ is the feature expectation vector estimated using the set of examples *D* and *N* the number of samples. Formally, this is equal to satisfy the following equation:

5 $$\begin{aligned} \sum _{t=0}^{T} \gamma ^t E[\phi (s_{t},a_{t})] = \frac{1}{\left| N \right| } \sum _{d \in D}^{}\sum _{t=0}^{T}\gamma ^t\phi (s^{d}_{t},a^{d}_{t}) \end{aligned}$$

A maximum-entropy approach has been proved to overcome some of the limitations pointed out by Abbeel and Ng (2004) regarding the ambiguity of policy and matching of feature counts and sub-optimality of solutions. Indeed, as it is a probabilistic approach is robust to noise and randomness in the actions of the expert. The maximum entropy in IRL finds a policy which is consistent with the examples that match feature expectations, without being committed to the constrains required by any of these samples (Ziebart et al. 2008). This is obtained by maximising $H(Y|X)=E_{P(Y,X)}[-log P(Y|X)]$, where Y are the predicted variables of the model and X are the side information about the problem, we don’t want to take into consideration, and the expectation is taken with respect to the join probability of Y and X. In IRL, X variables correspond to the sequence of states *S* generated by interacting with the environment and Y are sequences of actions in *A*. The causal entropy, in addition to the maximum entropy, states that there is a conditionally causal relationship between states and actions in a trajectory under a given policy. Formally, we have the following definition: $P_{\pi }(d)=P_{\pi }(A^{d} \parallel S^{d})=\prod _{t=1}^{n}\pi (A_{t}^{d}|S_{1:t}^{d}, A_{1:t-1}^{d})$, where $A^d$ and $S^d$ are actions and state in *d*, $A_{t}^{d}$ is the action at time *t* in *d*, and $A_{1:t-1}^{d}$ and $S_{1:t}^{d}$ are actions and states in *d* from time step 1 to *t*. In other words, $P_{\pi }(d)$ defines the joint distribution of actions in a trajectory *d* conditioned on the states in *d*. The causal entropy $H(A_{t} \parallel S_{t})$ is then defined as:

6 $$\begin{aligned} H(A_{t} \parallel S_{t})= \sum _{t=0}^{T}\gamma ^tE[-\log \pi (A_{t},S_{t})] \end{aligned}$$

which is the conditional entropy of the action sequence $A_{t}$, causally conditioned on the state sequence $S_{t}$.

The complete constrained optimisation problem is as follows:

7 $$\begin{aligned} \begin{aligned}&\text {find:} \max _{\pi }H(A^{h}\left| \right| S^{h}) \\&\text {subject to: } {\mathbf {f}}_{\pi }={\mathbf {f}}_{d} \\&\text {and: } \sum _{a \in A} \pi (s,a) = 1 \forall s \in S \\&\text {and: } \pi (s,a)\ge 0 \forall s \in S, \forall a \in A, \end{aligned} \end{aligned}$$

To solve this non-convex optimisation problem, the IRL is converted to an equivalent convex one by considering a Lagrangian relaxation of Eq. (7) and a dual problem formulation. By differentiating the Lagrangian with respect to the constraints of Eq. (7), the IRL problem takes the form of:

8 $$\begin{aligned} \pi _{\varTheta }(A_{t}| S_{t})= & {} e^{Q^\mathrm{{soft}}_{\pi _{\varTheta }}(A_{t},S_{t})-V^\mathrm{{soft}}_{\pi _{\varTheta }}(S_{t})} \end{aligned}$$

9 $$\begin{aligned} V^\mathrm{{soft}}_{\pi _{\varTheta }}(S_{t})= & {} \log \sum _{A_{t} \in A} e^{Q^\mathrm{{soft}}_{\pi _{\varTheta }}(A_{t},S_{t})} \end{aligned}$$

10 $$\begin{aligned} {Q^\mathrm{{soft}}_{\pi _{\varTheta }}(A_{t},S_{t})}= & {} R_{\varTheta }(S_{t},A_{t})\sum _{S^{'} \in S} P(S^{'}|A_{t}, S_{t})V^\mathrm{{soft}}_{\pi _{\varTheta }}(S_{t}) \end{aligned}$$

11 $$\begin{aligned} R_{\varTheta }(S_{t},A_{t})= & {} \sum _{k=1}^{K} \varTheta _{k} \phi _{k} (S_{t},A_{t}) \end{aligned}$$

with the definition of $\varTheta$ (dual parameters) and therefore of $\pi _{\varTheta }$ the MCE IRL problem can be solved by finding $\varTheta$. Once $\pi$ is computed for a give $\varTheta$, the latter is updated via gradient descent, considering that:

12 $$\begin{aligned} \nabla L_\mathrm{{Dual}}(\varTheta )={\mathbf {f}}_{\varTheta }-\sum _{(S,A) \in SxA} D_{S} {\hat{\pi }}_{\varTheta } (A|S) \phi (S,A) \end{aligned}$$

where $D_{S}$ is the visitation frequency of state *S*. Thus, to compute the gradient of the dual problem, it is necessary to compute the policy ${\hat{\pi }}_{\varTheta }$ and $D_{S}$. The algorithms for computing the gradient as well as the soft value function and the expected state visitation frequency are detailed in Ziebart’s thesis (Ziebart 2010).

## Footnotes

1 https://erdogant.github.io/bnlearn/.

2 https://github.com/aandriella/CARESSER.

3 http://www.iri.upc.edu/groups/perception/CARESSER.

4 http://www.iri.upc.edu/groups/perception/CARESSER.
